# Nicotinamide Riboside Supplementation Benefits in Patients With Werner Syndrome: A Double‐Blind Randomized Crossover Placebo‐Controlled Trial

**DOI:** 10.1111/acel.70093

**Published:** 2025-06-03

**Authors:** Mayumi Shoji, Hisaya Kato, Masaya Koshizaka, Hiyori Kaneko, Yusuke Baba, Takahiro Ishikawa, Naoya Teramoto, Daisuke Kinoshita, Ayano Yamaguchi, Yukari Maeda, Yosuke Inaba, Yuki Shiko, Yoshihito Ozawa, Vilhelm A. Bohr, Yoshiro Maezawa, Koutaro Yokote

**Affiliations:** ^1^ Department of Endocrinology, Hematology and Gerontology Chiba University Graduate School of Medicine Chiba Japan; ^2^ Division of Diabetes, Department of Medicine, Metabolism and Endocrinology Chiba University Hospital Chiba Japan; ^3^ Clinical Research Center, Chiba University Hospital Chiba Japan; ^4^ Center for Preventive Medical Science, Chiba University Chiba Japan; ^5^ Department of General Medical Science Chiba University Graduate School of Medicine Chiba Japan; ^6^ Department of Metabolism and Endocrinology Eastern Chiba Medical Center Togane Japan; ^7^ Laboratory of Molecular Gerontology National Institute on Aging, NIH Baltimore Maryland USA; ^8^ Center for Healthy Aging University of Copenhagen Copenhagen Denmark

**Keywords:** arterial stiffness, cardio–ankle vascular index, creatinine, intractable skin ulcers, metabolome, nicotinamide riboside, Werner syndrome

## Abstract

Werner syndrome (WS) is a rare hereditary progeroid syndrome caused by mutations in the *WRN* gene. Patients frequently develop various age‐associated diseases prematurely, often leading to early mortality (≤ 60 years of age). Depletion of nicotinamide adenine dinucleotide (NAD)^+^ has been reported in patients with WS, suggesting a key role in the pathogenesis of WS. NAD^+^ supplementation may improve the condition of WS and other accelerated aging diseases. Therefore, we conducted a double‐blind, randomized, crossover, placebo‐controlled trial in patients with WS to evaluate the safety and efficacy of the NAD^+^ precursor, nicotinamide riboside (NR). NR (1000 mg) or placebo capsules were self‐administered once daily for 26 weeks, followed by a crossover to the opposite arm for another 26 weeks. The primary endpoint was the safety of NR. Secondary endpoints included NAD^+^ levels in plasma, number, and size of skin ulcers, blood examinations, sarcopenia, heel pad thickness, cardio–ankle vascular index (CAVI), and ankle–brachial index. The exploratory endpoints involved metabolome profiles of plasma. No serious adverse events were observed during NR treatment. Importantly, CAVI improved, the skin ulcer area decreased, and heel pad thinning showed a declining trend. Metabolomic analysis revealed a significant decrease in blood creatinine. NR treatment significantly improved arterial stiffness, as indicated by CAVI, and likely suppressed renal functional decline in patients with WS. Therefore, NR may be beneficial for preventing atherosclerosis, skin ulcers, and kidney dysfunction in patients with WS.

## Graphical abstract

Nicotinamide riboside improved arterial stiffness (cardio–ankle vascular index), reduced skin ulcer area, and showed a trend toward mitigating heel pad thinning in patients with Werner syndrome, with no serious adverse events. Metabolomic analysis revealed a significant decrease in creatinine, suggesting a potential suppression of renal functional decline.

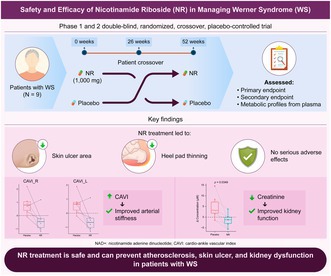

## 1 Introduction

Werner syndrome (WS) is a rare, hereditary progeroid syndrome characterized by defects in DNA metabolism and mitochondrial function (Oshima et al. 2017; Yokote et al. 2017). It is caused by mutations in *WRN*, which is involved in DNA replication, repair, and telomere maintenance (Croteau et al. 2014). The prevalence of WS is estimated to be nine cases per million people in Japan (Kato and Maezawa 2022). WS is frequently accompanied by metabolic disorders such as diabetes, dyslipidemia, cardiovascular disease, and malignancy. Patients with WS also exhibit characteristic features such as short stature, low body weight, sarcopenia, and diminished subcutaneous tissue. Their skin is typically atrophic and tight. Clavus, calluses, and intractable ulcers on the feet are also frequently observed. WS is a devastating disease that leads to severe skin ulcers, malignant tumors, and ultimately premature death at a mean age of 59 years (Kato et al. 2022). Currently, there is no standard treatment for this disease (Koshizaka et al. 2020; Maeda et al. 2023; Shamanna et al. 2017).

Nicotinamide adenine dinucleotide (NAD)^+^ is a vital coenzyme in all living cells, essential for maintaining intracellular NAD^+^ levels to support cellular and metabolic processes, including adenosine triphosphate (ATP) production and DNA repair. In mammals, NAD^+^ can be synthesized from tryptophan, nicotinic acid, nicotinamide, and nicotinamide riboside (NR) (Imai and Guarente 2014; Yang et al. 2023). NR was first identified as a vitamin (Bieganowski and Brenner 2004) and later recognized as one of three vitamin precursors of NAD^+^ (Bogan and Brenner 2008). It is present in trace amounts in milk and yeast.

Previous studies have examined NAD^+^ precursors in healthy individuals, patients with obesity, and older adults (Tables S1 and S2). NR supplementation has been shown to increase NAD^+^ levels, provide neuroprotection in neurodegenerative diseases (Vaur et al. 2017; Hou et al. 2018), and reduce the incidence of heart failure in dilated cardiomyopathy (Diguet et al. 2018).

In obese mice, hepatic nicotinamide adenine dinucleotide phosphate (NADPH) is impaired; however, NR administration has been shown to restore hepatic NADPH levels, leading to improvements in blood glucose regulation, fatty liver, and the prevention of diabetic neuropathy (Trammell et al. 2016a). In humans, NR safely increases NAD^+^ without reported side effects (Conze et al. 2019), and numerous clinical trials have been conducted in recent years.

In older adults, NR administration has been associated with decreased circulating levels of inflammatory cytokines, including IL‐6, IL‐5, IL‐2, and TNF‐α (Elhassan et al. 2019). In patients with systemic lupus erythematosus, NR reduced relative mRNA expression of inflammatory cytokines IFN‐β and CXCL10 (Wu et al. 2022). Similarly, in heart failure patients, NR downregulated the gene expression of NLRP3 and inflammatory cytokines IL‐1B, IL‐6, IL‐18 (Zhou et al. 2020) and TNF‐α (Wang et al. 2022). In Parkinson's disease patients, NR reduced serum levels of inflammatory cytokines VEGF and GDF15 and cerebrospinal fluid levels of G‐CSF, IL‐7, IL‐1RA, and CCL4 (Brakedal et al. 2022). Furthermore, in patients with nonalcoholic fatty liver disease (NAFLD), administration of a compound metabolic activator containing NR led to improvements in liver enzyme levels and hepatic steatosis (Zeybel et al. 2022). Collectively, these findings indicate that NR mitigates chronic inflammation across various diseases in humans.

In patients with WS, impaired mitophagy and NAD^+^ depletion have been reported (Fang et al. 2019). WS model systems have hyperPARylation (Fang et al. 2019), which is excessive poly(ADP‐ribosyl)ation of DNA damage response proteins, primarily mediated by the enzyme poly ADP‐ribose polymerase 1 (PARP1) in response to DNA damage. Since NAD is a substrate for this enzyme, this hyperPARylation may be at least partially responsible for the NAD depletion observed in WS. WRN, a key regulator of NAD^+^ metabolism, modulates the transcription of nicotinamide nucleotide adenylyl transferase 1 (NMNAT1), an NAD^+^ biosynthetic enzyme (Fang et al. 2019). NAD^+^ repletion restores the NAD^+^ metabolic profile and improves mitochondrial function, significantly extending lifespan and delaying features of accelerated aging, including stem cell dysfunction, in *Caenorhabditis elegans* and *Drosophila melanogaster* models of WS (Fang et al. 2019). These findings suggest that accelerated aging in WS is mediated, at least in part, by mitochondrial dysfunction and impaired mitophagy and that increasing cellular NAD^+^ levels may counteract WS phenotypes.

Currently, no fundamental treatment exists for WS, and disease management is limited to symptomatic therapies targeting refractory skin ulcers, sarcopenia, and atherosclerotic disease (Kubota et al. 2021; Kuzuya et al. 2021; Tsukamoto et al. 2021). However, these treatments have limited efficacy, underscoring the need for more effective approaches. Chronic inflammation contributes to metabolic disorders such as fatty liver and diabetes, as well as to complications including skin ulcers and atherosclerotic disease, all of which are major concerns in WS.

Given NR's potential to mitigate sarcopenia, metabolic disorders, and atherosclerotic disease, it may slow the progression of aging in WS. However, no studies have assessed NR administration in patients with WS. Therefore, we aimed to evaluate the safety and efficacy of NR in patients with WS through a double‐blind, randomized, crossover, placebo‐controlled trial.

## 2 Results

### 2.1 Patient Characteristics

**FIGURE 1:**
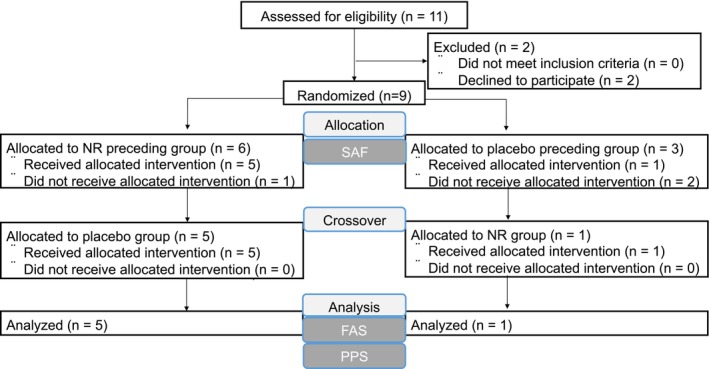
A flow chart showing the CONSORT protocol. FAS, full analysis set; NR, nicotinamide riboside; PPS, per‐protocol set; SAF, safety analysis set.

Eleven patients with WS were enrolled in the study, and nine were randomized (Figure 1). Patient characteristics are shown in Table 1. The average age of the patients was 47 ± 8.1 years, and four patients (44.4%) were male. Comorbidities included hypertension in two patients (22.2%), dyslipidemia in seven patients (77.8%), and diabetes mellitus in six patients (66.7%). Three patients (33.3%) had intractable skin ulcers. None of the patients had undergone limb amputation. Three patients (33.3%) had parents who were consanguineously married. All patients underwent genetic testing and were diagnosed with mutations in *WRN*, with 88.9% of these mutations corresponding to a type 4 mutation. Regarding the Japanese WRN gene, three types of mutations, known as type 1, type 4, and type 6, account for 85.7% of the total alleles (Yamaga et al. 2017a). The type 4 mutation, in which guanine, the base immediately preceding exon 26, is mutated to cytosine (c. 319‐1G > C), accounts for 72.1% of Japanese WS cases (Yamaga et al. 2017a).

**TABLE 1:** Patient characteristics at baseline.

	FAS/PPS			SAF		
	NR administered prior (*n* = 5) mean ± SD	Placebo administered prior (*n* = 1) mean ± SD	Total (*n* = 6) mean ± SD	NR administered prior (*n* = 6) mean ± SD	Placebo administered prior (*n* = 3) mean ± SD	Total (*n* = 9) mean ± SD
Age (years)	44.2 ± 4.87	45	44.3 ± 4.37	43.8 ± 4.45	53.3 ± 11.2	47.0 ± 8.12
Female, *n* (%)	2 (40)	1 (100)	3 (50)	3 (50)	2 (66.7)	5 (55.6)
Height (cm)	*n* = 5 158.3 ± 10.4	*n* = 1 152	*n* = 6 157.3 ± 9.7	*n* = 6 157.4 ± 9.7	*n* = 2 156.3 ± 6.1	*n* = 8 157.1 ± 8.5
Body weight (kg)	*n* = 5 38.9 ± 10.5	*n* = 1 50	*n* = 6 40.8 ± 10.4	*n* = 6 39.7 ± 9.5	*n* = 2 54.9 ± 6.9	*n* = 8 43.5 ± 11.0
BMI (kg/m^2^)	*n* = 5 15.3 ± 2.46	*n* = 1 21.6	*n* = 6 16.4 ± 3.40	*n* = 6 15.9 ± 2.62	*n* = 2 22.4 ± 1.09	*n* = 8 17.5 ± 3.77
Waist circumference (cm)	72.0 ± 10.7	84	74.0 ± 10.8	72.1 ± 9.6	88.7 ± 5.0	77.6 ± 11.5
Systolic blood pressure (mmHg)	107.6 ± 19.8	147	114.2 ± 24.0	112.8 ± 21.9	127.7 ± 16.9	117.8 ± 20.6
Diastolic blood pressure (mmHg)	61.0 ± 9.6	84	64.8 ± 12.7	63.3 ± 10.3	65.7 ± 17.2	64.1 ± 11.9
Pulse (bpm)	*n* = 5 75.0 ± 7.7	*n* = 1 73	*n* = 6 74.7 ± 7.0	*n* = 6 77.7 ± 9.5	*n* = 2 84.5 ± 16.3	*n* = 8 79.4 ± 10.6
Right grip strength (kg)	21.1 ± 7.2	11.7	19.6 ± 7.5	21.2 ± 6.5	13.8 ± 10.1	18.8 ± 8.1
Left grip strength (kg)	*n* = 4 19.1 ± 7.2	*n* = 1 10.4	*n* = 5 17.4 ± 7.4	*n* = 5 19.8 ± 6.4	*n* = 3 12.6 ± 9.8	*n* = 8 17.1 ± 8.1
Walking speed (m/s)	*n* = 4 1.23 ± 0.32	*n* = 1 1.43	*n* = 5 1.27 ± 0.29	*n* = 5 1.27 ± 0.29	*n* = 2 0.84 ± 0.83	*n* = 7 1.14 ± 0.46
Visceral fat area at umbilical height (cm^2^)	86.1 ± 79.6	107.31	89.6 ± 71.7	84.6 ± 71.3	114.4 ± 43.7	94.5 ± 62.2
Skeletal muscle index (kg/m^2^)	4.03 ± 0.93	5.16	4.22 ± 0.95	4.19 ± 0.92	3.66 ± 1.45	4.01 ± 1.06
Right heel pad thickness (mm)	6.38 ± 1.40	7.5	6.57 ± 1.33	6.90 ± 1.79	5.37 ± 1.87	6.39 ± 1.86
Left heel pad thickness (mm)	6.72 ± 2.06	7.6	6.87 ± 2.76	6.93 ± 2.79	7.77 ± 2.75	7.21 ± 2.63
Right CAVI	8.12 ± 1.10	7.8	8.07 ± 1.00	8.02 ± 1.02	8.80 ± 1.73	8.28 ± 1.25
Left CAVI	*n* = 5 8.24 ± 1.07	*n* = 1 8.2	*n* = 6 8.23 ± 0.96	*n* = 6 8.08 ± 1.03	*n* = 2 7.95 ± 0.35	*n* = 8 8.05 ± 0.89
Right ABI	1.01 ± 0.15	1.01	1.01 ± 0.13	1.04 ± 0.16	1.04 ± 0.21	1.04 ± 0.16
Left ABI	*n* = 5 1.01 ± 0.09	*n* = 1 1.06	*n* = 6 1.02 ± 0.08	*n* = 6 1.02 ± 0.08	*n* = 2 1.08 ± 0.03	*n* = 8 1.04 ± 0.08
Numerical rating scale	4.2 ± 1.8	1	3.7 ± 2.1	3.7 ± 2.1	5.7 ± 4.5	4.3 ± 3.0
Diabetes mellitus, *n* (%)	3 (60)	0 (0)	3 (50)	4 (66.7)	2 (66.7)	6 (66.7)
Dyslipidemia, *n* (%)	4 (80)	1 (100)	5 (83.3)	5 (83.3)	2 (66.7)	7 (77.8)
Hypertension, *n* (%)	1 (20)	0 (0)	1 (16.7)	1 (16.7)	1 (33.3)	2 (22.2)
Fatty liver, *n* (%)	2 (40)	1 (100)	3 (50)	3 (50)	2 (66.7)	5 (55.6)
Cerebral hemorrhage, *n* (%)	1 (20)	0 (0)	1 (16.7)	1 (16.7)	0 (0)	1 (11.1)
Cerebral infarction, *n* (%)	0 (0)	0 (0)	0 (0)	0 (0)	0 (0)	0 (0)
Angina pectoris or myocardial infarction, *n* (%)	1 (20)	0 (0)	1 (16.7)	1 (16.7)	0 (0)	1 (11.1)
Arteriosclerosis obliterans, *n* (%)	2 (40)	0 (0)	2 (33.3)	2 (33.3)	1 (33.3)	3 (33.3)
Malignancy, *n* (%)	0 (0)	0 (0)	0 (0)	0 (0)	0 (0)	0 (0)
Hypothyroidism, *n* (%)	4 (80)	1 (100)	5 (83.3)	5 (83.3)	1 (33.3)	6 (66.7)
Barthel index	96 ± 6.5	100	96.7 ± 6.1	96.7 ± 6.1	71.7 ± 49.1	88.3 ± 28.0
Gray hair or baldness	5 (100)	1 (100)	6 (100)	6 (100)	3 (100)	9 (100)
Bilateral cataract	5 (100)	1 (100)	6 (100)	6 (100)	3 (100)	9 (100)
Skin atrophy or hardening	5 (100)	1 (100)	6 (100)	6 (100)	3 (100)	9 (100)
Skin ulcer	3 (60)	0 (0)	3 (50)	3 (50)	2 (66.7)	5 (55.6)
Soft tissue calcification	5 (100)	1 (100)	6 (100)	6 (100)	3 (100)	9 (100)
Bird‐like face	5 (100)	0 (0)	5 (83.3)	6 (100)	2 (66.7)	8 (88.9)
High‐pitched voice	5 (100)	0 (0)	5 (83.3)	5 (83.3)	2 (66.7)	7 (77.8)
Limb amputation, *n* (%)	0 (0)	0 (0)	0 (0)	0 (0)	0 (0)	0 (0)
Family history of Werner syndrome, *n* (%)	0 (0)	0 (0)	0 (0)	0 (0)	1 (33.3)	1 (11.1)
Consanguineous marriage, *n* (%)	1 (20)	0 (0)	1 (16.7)	1 (16.7)	2 (66.7)	3 (33.3)
Genetic testing, *n* (%)	5 (100)	1 (100)	6 (100)	6 (100)	3 (100)	9 (100)
***WRN* mutation type, *n* (%)**						
Mutation 4/4	2 (40)	1 (100)	3 (50.0)	2 (33.3)	3 (100)	5 (55.5)
Mutation 4/6	0 (0)	0 (0)	0 (0)	1 (16.7)	0 (0)	1 (11.1)
Mutation 4/19	1 (20)	0 (0)	1 (16.7)	1 (16.7)	0 (0)	1 (11.1)
Mutation 4/23	1 (20)	0 (0)	1 (16.7)	1 (16.7)	0 (0)	1 (11.1)
Mutation 20/21	1 (20)	0 (0)	1 (16.7)	1 (16.7)	0 (0)	1 (11.1)
**Blood and urine test**						
WBC (/μL)	5960 ± 971	5100	5817 ± 937	6350 ± 1291	8200 ± 3503	6967 ± 2228
RBC (×10^4^/μL)	418 ± 37	472	427 ± 40	422 ± 34	385 ± 80	410 ± 52
Hemoglobin (g/dL)	12.8 ± 1.1	15.0	13.2 ± 1.3	12.9 ± 1.0	11.5 ± 3.2	12.4 ± 1.9
Hematocrit (%)	38.5 ± 3.3	44.3	39.5 ± 3.8	39.0 ± 3.2	36.2 ± 7.5	38.1 ± 4.7
Platelet (×10^4^/μL)	20.9 ± 7.1	23.1	21.3 ± 6.4	21.1 ± 6.4	32.7 ± 11.3	25.0 ± 9.5
AST (U/L)	36.2 ± 19.6	30	35.2 ± 17.7	36.0 ± 17.6	30.3 ± 1.5	34.1 ± 14.2
ALT (U/L)	37.8 ± 16.6	39	38.0 ± 14.9	41.3 ± 17.2	31.7 ± 13.6	38.1 ± 16.0
LDH (U/L)	190 ± 57.3	219	195 ± 52.6	192 ± 51.5	190 ± 39.5	191 ± 45.3
ALP (U/L)	83.4 ± 17.2	106	87.2 ± 17.9	86.2 ± 16.8	85.3 ± 29.1	85.9 ± 19.7
γGTP (U/L)	62.0 ± 32.5	97	67.8 ± 32.4	63.0 ± 63.0	55.0 ± 39.3	60.3 ± 30.6
CPK (U/L)	133 ± 124	122	131 ± 111	125 ± 112	84.0 ± 55.7	112 ± 95.3
T‐bilirubin (mg/dL)	0.62 ± 0.23	0.6	0.62 ± 0.20	0.60 ± 0.21	0.43 ± 0.15	0.54 ± 0.20
C‐bilirubin (mg/dL)	0.08 ± 0.04	0.1	0.08 ± 0.04	0.07 ± 0.05	0.07 ± 0.06	0.07 ± 0.05
Total protein (g/dL)	7.56 ± 0.51	7.3	7.52 ± 0.47	7.50 ± 0.48	8.07 ± 0.93	7.69 ± 0.66
Serum albumin (g/dL)	4.44 ± 0.18	4.5	4.45 ± 0.16	4.48 ± 0.19	3.53 ± 0.91	4.17 ± 0.67
Uric acid (mg/dL)	5.08 ± 1.75	4.8	5.03 ± 1.57	5.37 ± 1.72	4.77 ± 0.35	5.17 ± 1.40
Na (mmol/L)	142 ± 1.8	140	141 ± 1.8	142 ± 1.6	138 ± 2.5	140 ± 2.7
K (mmol/L)	4.04 ± 0.17	3.9	4.02 ± 0.16	4.05 ± 0.15	4.07 ± 0.67	4.06 ± 0.35
Cl (mmol/L)	106 ± 1.6	105	106 ± 1.5	106 ± 1.7	103 ± 2.1	105 ± 2.3
Total cholesterol (mg/dL)	182 ± 36.0	236	191 ± 39.1	185 ± 33.3	196 ± 34.9	189 ± 32.0
Triglyceride (mg/dL)	133 ± 68.3	109	129 ± 61.9	137 ± 62.0	121 ± 10.1	132 ± 50.0
LDL‐cholesterol (mg/dL)	94.8 ± 28.9	154	105 ± 35.4	98.0 ± 27.0	118 ± 32.1	105 ± 28.5
HDL‐cholesterol (mg/dL)	68.8 ± 14.5	66	68.3 ± 13.0	68.3 ± 13.0	53.7 ± 10.8	63 ± 13.7
Serum glucose (mg/dL)	111 ± 13.1	98	109 ± 12.9	114 ± 14.0	132 ± 29.4	120 ± 20.4
HbA1c (%)	6.12 ± 1.34	5.2	5.97 ± 1.26	6.15 ± 1.20	6.47 ± 1.25	6.26 ± 1.15
CRP (mg/dL)	0.17 ± 0.21	0.15	0.16 ± 0.19	0.17 ± 0.19	8.21 ± 11.83	2.85 ± 7.15
NAD^+^ (unit)	0.06 ± 0.02	0.04	0.05 ± 0.02	0.06 ± 0.01	0.11 ± 0.08	0.08 ± 0.06
BUN (mg/dL)	17.2 ± 6.1	10	16.0 ± 6.2	17.0 ± 5.5	14.0 ± 3.6	16.0 ± 4.9
Serum creatinine (mg/dL)	0.64 ± 0.14	0.53	0.62 ± 0.13	0.66 ± 0.14	0.67 ± 0.16	0.66 ± 0.14
eGFR (mL/min/1.73 m^2^)	99.4 ± 25.8	96.3	98.9 ± 23.1	93.6 ± 27.1	81.1 ± 13.4	89.4 ± 23.3
Urine albumin (mg/gCr)	9.6 ± 3.5	5	8.8 ± 3.7	10.7 ± 4.1	56.6 ± 86.1	26.0 ± 48.9
Urine NAG (U/L)	6.8 ± 6.6	3.8	6.3 ± 6.0	6.7 ± 5.9	23.8 ± 17.6	12.4 ± 13.1
β2‐microglobulin (mg/L)	114 ± 99.7	73	108 ± 90.7	129 ± 96.2	7276 ± 11,746	2511 ± 6875
L‐FABP (μg/gCr)	3.48 ± 0.55	2.52	3.32 ± 0.63	3.41 ± 0.53	97.5 ± 156	34.8 ± 91.3
Kim‐1 (ng/mL)	0.74 ± 0.93	0.87	0.76 ± 0.83	0.77 ± 0.84	1.43 ± 0.71	0.99 ± 0.82

Abbreviations: γGTP, gamma‐glutamyl transferase; ABI, ankle–brachial index; ALP, alkaline phosphatase; ALT, alanine aminotransferase; AST, aspartate aminotransferase; BUN, blood urea nitrogen; C‐bilirubin, conjugated bilirubin; CPK, creatine phosphokinase; CRP, C‐reactive protein; eGFR, estimated glomerular filtration rate; FAS, full analysis set; HbA1c, HbA1c, glycated hemoglobin; HDL, high‐density lipoprotein; IQR, interquartile range; Kim‐1, kidney injury molecule‐1; LDH, lactate dehydrogenase; LDL, low‐density lipoprotein; L‐FABP, liver‐type fatty acid‐binding protein; mutation 19, c.2344_2351delGGTGAACT; mutation 20, c.2630G > C; mutation 21, c.2630G > T; mutation 23, c.3383 + 1G > T, CAVI, cardio–ankle vascular index; mutation 4, c.3139‐1G > C; mutation 6, c.1105C > T; NA, not available; NAD, nicotinamide adenine dinucleotide; NAG, N‐acetyl‐β‐D‐glucosaminidase; NR, nicotinamide riboside; PPS, per‐protocol set; RBC, red blood cell; SAF, safety analysis set; SD, standard deviation; T‐bilirubin, total bilirubin; WBC, white blood cell.

### 2.2 Primary Endpoint: Safety of Nicotinamide Riboside

During the NR phase, no moderate or severe adverse events were reported. Seven mild adverse events occurred during the NR phase: pain, slightly abnormal liver function (increased aspartate aminotransferase [AST]), subcutaneous hemorrhage, hyperkeratosis, pruritic skin rash, skin ulcers, and hypotension (Supplementary Table 3). By contrast, 12 adverse events occurred during the placebo phase, including increased tear flow, diarrhea, pneumonia, COVID‐19 infection, chilblains, synovial fluid cysts, frequent urination, renal enlargement, renal mass, alopecia, skin pain, and pruritus.

### 2.3 Secondary Endpoints: NAD ^+^ Levels in Patients With WS

**FIGURE 2:**
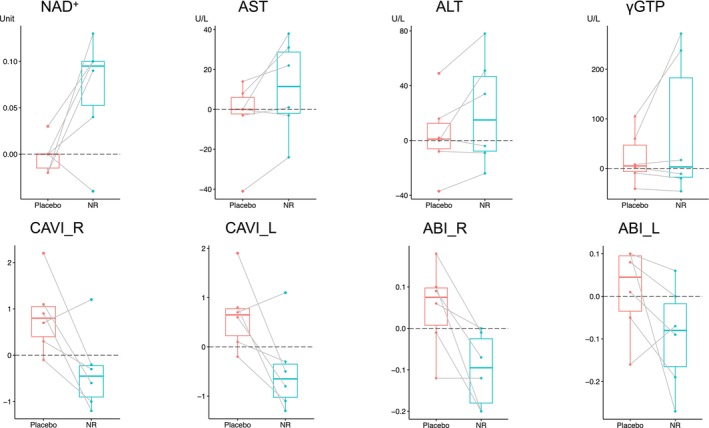
Changes in NAD^+^, AST, ALT, γGTP, CAVI, and ABI. Red bar and box show changes in the placebo phase, blue bar and box show changes in the NR treatment phase. Medium bars show the mean, top and bottom bars of the box show the standard deviation. γGTP, gamma‐glutamyl transferase; ABI, ankle–brachial index; ALT, alanine aminotransferase; AST, aspartate aminotransferase; CAVI, cardio–ankle vascular index; NAD, nicotinamide adenine dinucleotide.

Plasma NR levels were low at baseline and during the placebo phase but increased significantly in the NR phase (change at 24 weeks: NR vs. placebo: 5.4e−05 ± 3.2e−05 units vs. 5.6e−06 ± 8.4e−06 units, *p* = 0.00239*). The average baseline NAD^+^ level, as determined via metabolomic analysis, was 0.050 ± 0.020 units. As expected, NAD^+^ levels increased significantly following NR treatment (change at 24 weeks NR vs. placebo: 0.070 ± 0.061 units vs. −0.002 ± 0.018 units, *p* = 0.045*) (Table 2, Figure 2). These findings confirm that NR treatment effectively increases plasma NAD^+^ and NR levels in patients with WS.

**TABLE 2:** Changes in clinical parameters from baseline in each group.

Biometric measurements	NR (*n* = 6) mean ± SD median (IQR)	Placebo (*n* = 6) mean ± SD median (IQR)	*p*
Systolic blood pressure (mmHg) *n* = 6	4.5 ± 20.8 −4.0 (−9.0, 13.0)	1.7 ± 13.8 −2.0 (−3.0, 2.0)	0.586
Diastolic blood pressure (mmHg) *n* = 6	3.0 ± 12.7 4.0 (−8.0, 8.0)	6.0 ± 10.5 2.0 (0.0, 11.0)	0.537
Right grip strength (kg) *n* = 6	−0.6 ± 2.3 −0.5 (−2.5, 1.4)	−1.2 ± 2.7 −0.4 (−0.8, −0.1)	0.563
Left grip strength (kg) *n* = 5	−1.2 ± 1.7 −2.3 (−2.4, 0.4)	−0.2 ± 2.0 0.5 (−1.1, 0.9)	0.505
Walking speed (m/s) *n* = 5	0.0 ± 0.2 −0.0 (−0.1, 0.1)	−0.0 ± 0.2 −0.0 (−0.1, 0.0)	0.584
Visceral fat area at umbilical height (cm^2^) *n* = 6	−18.3 ± 47.9 5.4 (−44.5, 8.8)	−0.5 ± 48.4 17.5 (−8.9, 22.6)	0.271
Skeletal muscle index (kg/m^2^) *n* = 6	0.0 ± 0.1 −0.0 (−0.0, 0.1)	0.0 ± 0.3 −0.1 (−0.2, 0.2)	0.671
Right heel pad thickness (mm) *n* = 6	−0.2 ± 1.2 −0.5 (−1.0, 0.4)	−0.7 ± 0.9 −0.8 (−1.4, −0.1)	0.273
Left heel pad thickness (mm) *n* = 6	0.1 ± 1.6 0.8 (−0.8, 1.2)	−0.7 ± 2.1 −0.2 (−0.6, 0.4)	0.060
Right CAVI *n* = 6	−0.4 ± 0.9 −0.5 (−1.0, −0.2)	0.9 ± 0.8 0.8 (0.3, 1.1)	0.042
Left CAVI *n* = 6	−0.5 ± 0.9 −0.6 (−1.1, −0.3)	0.7 ± 0.7 0.7 (0.1, 0.8)	0.046
Right ABI *n* = 6	−0.1 ± 0.1 −0.1 (−0.2, −0.0)	0.1 ± 0.1 0.1 (−0.0, 0.1)	0.018
Left ABI *n* = 6	−0.1 ± 0.1 −0.1 (−0.2, 0.0)	0.0 ± 0.1 0.0 (−0.1, 0.1)	0.145
Numerical Rating Scale *n* = 6	0.7 ± 3.1 1.5 (0.0, 2.0)	−1.0 ± 2.4 −0.5 (−2.0, 0.0)	0.175
**Blood and urine test**			
WBC (/μL) *n* = 6	250 ± 979 300 (−400, 1100)	550 ± 1211 250 (−100, 800)	0.427
RBC (×10^4^/μL) *n* = 6	6.667 ± 21.125 4.000 (4.000, 24.000)	3.833 ± 33.654 −3.500 (−18.000, 7.000)	0.833
Hemoglobin (g/dL) *n* = 6	0.317 ± 0.402 0.500 (0.100, 0.600)	0.000 ± 0.959 −0.100 (−0.400, 0.300)	0.479
Hematocrit (%) *n* = 6	1.050 ± 1.472 1.750 (0.100, 2.100)	0.700 ± 2.808 0.350 (−1.400, 1.500)	0.799
Platelet (×10^4^/μL) *n* = 6	−0.200 ± 1.691 0.150 (−1.800, 1.200)	0.300 ± 2.263 −0.200 (−1.100, 1.200)	0.697
AST (U/L) *n* = 6	10.83 ± 23.54 11.50 (−3.00, 31.00)	−3.67 ± 19.34 0.00 (−3.00, 8.00)	0.061
ALT (U/L) *n* = 6	21.00 ± 39.67 15.00 (−9.00, 51.00)	3.667 ± 28.33 1.00 (−8.00, 16.00)	0.097
LDH (U/L) *n* = 6	16.500 ± 19.644 21.500 (0.000, 28.000)	−7.833 ± 45.775 2.500 (1.000, 7.000)	0.314
ALP (U/L) *n* = 6	17.833 ± 27.651 6.000 (0.000, 45.000)	14.500 ± 10.747 9.500 (7.000, 20.000)	0.678
γGTP (U/L) *n* = 6	75.00 ± 141.28 3.00 (−20.00, 238.00)	20.83 ± 52.63 5.00 (−9.00, 60.00)	0.223
CPK (U/L) *n* = 6	56.500 ± 62.292 29.500 (16.000, 84.000)	−27.833 ± 100.280 4.000 (−13.000, 29.000)	0.250
T‐bilirubin (mg/dL) *n* = 6	0.000 ± 0.063 0.000 (0.000, 0.000)	−0.050 ± 0.152 −0.050 (−0.200, 0.000)	0.415
C‐bilirubin (mg/dL) *n* = 6	0.017 ± 0.041 0.000 (0.000, 0.000)	0.000 ± 0.063 0.000 (0.000, 0.000)	0.363
Total protein (g/dL) *n* = 6	0.133 ± 0.413 −0.050 (−0.100, 0.300)	0.250 ± 0.152 0.250 (0.200, 0.400)	0.470
Serum albumin (g/dL) *n* = 6	0.117 ± 0.279 0.000 (−0.100, 0.300)	0.167 ± 0.294 0.300 (−0.100, 0.400)	0.695
Uric acid (mg/dL) *n* = 6	0.183 ± 0.685 0.150 (−0.100, 0.400)	0.800 ± 0.938 0.500 (0.000, 1.700)	0.088
Na (mmol/L) *n* = 6	−1.833 ± 0.983 −1.500 (−3.000, −1.000)	−0.833 ± 1.169 −1.000 (−2.000, 0.000)	0.076
K (mmol/L) *n* = 6	0.083 ± 0.232 0.050 (−0.100, 0.300)	0.350 ± 0.274 0.300 (0.200, 0.500)	0.140
Cl (mmol/L) *n* = 6	−1.667 ± 1.211 −1.500 (−3.000, −1.000)	−0.833 ± 1.472 −0.500 (−2.000, 0.000)	0.093
Total cholesterol (mg/dL) *n* = 6	21.00 ± 27.44 29.50 (−8.00, 46.00)	19.00 ± 20.07 24.50 (19.00, 33.00)	0.852
Triglyceride (mg/dL) *n* = 6	6.833 ± 39.45 1.500 (−16.000, 48.000)	0.833 ± 57.95 −0.500 (−9.000, 52.00)	0.880
LDL‐cholesterol (mg/dL) *n* = 6	11.33 ± 16.40 14.00 (4.000, 19.00)	15.00 ± 19.43 15.00 (4.000, 32.00)	0.722
HDL‐cholesterol (mg/dL) *n* = 6	10.83 ± 15.16 16.00 (−4.00, 19.00)	3.167 ± 9.517 4.50 (−8.00, 12.00)	0.194
Serum glucose (mg/dL) *n* = 6	−1.833 ± 16.53 0.000 (−6.000, 12.00)	8.333 ± 14.88 5.000 (−1.000, 5.000)	0.333
HbA1c (%) *n* = 6	−0.300 ± 0.961 −0.000 (−0.200, 0.100)	−0.217 ± 0.893 0.050 (−0.100, 0.300)	0.517
CRP (mg/dL) *n* = 6	0.590 ± 1.670 −0.010 (−0.030, 0.040)	−0.042 ± 0.098 −0.010 (−0.100, 0.020)	0.382
NAD^+^ (unit) *n* = 6	0.070 ± 0.061 0.095 (0.040, 0.100)	−0.002 ± 0.018 0.000 (−0.020, 0.000)	0.045
BUN (mg/dL) *n* = 6	−1.333 ± 3.933 −1.000 (−4.000, 2.000)	−0.333 ± 6.683 −1.000 (−5.000, 2.000)	0.593
Serum creatinine (mg/dL) *n* = 6	−0.002 ± 0.047 −0.015 (−0.030, 0.050)	0.092 ± 0.146 0.020 (0.020, 0.110)	0.249
eGFR (mL/min/1.73 m^2^) *n* = 6	0.467 ± 7.388 2.600 (−5.200, 7.000)	−10.733 ± 11.144 −5.350 (−22.000, −3.800)	0.088
Urine albumin (mg/gCr) *n* = 6	13.52 ± 31.27 2.050 (−0.800, 8.500)	16.07 ± 36.09 4.150 (0.900, 6.200)	0.363
Urine NAG (U/L) *n* = 6	−0.783 ± 3.857 −1.250 (−1.600, 1.600)	−3.450 ± 5.373 −2.750 (−8.400, −0.900)	0.349
β2‐microglobulin (mg/L) *n* = 6	25.83 ± 165.3 −19.50 (−37.00, 40.00)	395.8 ± 1093 −11.50 (−58.00, 25.00)	0.378
L‐FABP (μg/gCr) *n* = 6	1.040 ± 1.313 1.100 (0.055, 2.025)	1.567 ± 4.640 0.330 (−1.060, 1.220)	0.443
Kim‐1 (ng/mL) *n* = 6	−0.090 ± 0.716 −0.303 (−0.421, −0.093)	1.964 ± 6.078 −0.218 (−0.789, −0.093)	0.393

Abbreviations: γGTP, gamma‐glutamyl transferase; ABI, ankle–brachial index; ALP, alkaline phosphatase; ALT, alanine aminotransferase; AST, aspartate aminotransferase; BUN, blood urea nitrogen; CAVI, cardio–ankle vascular index; C‐bilirubin, conjugated bilirubin; CPK, creatine phosphokinase; CRP, C‐reactive protein; eGFR, estimated glomerular filtration rate; HbA1c, HbA1c, glycated hemoglobin; HDL, high‐density lipoprotein; IQR, interquartile range; Kim‐1, kidney injury molecule‐1; LDH, lactate dehydrogenase; LDL, low‐density lipoprotein; L‐FABP, liver‐type fatty acid‐binding protein; NA, not available; NAD, nicotinamide adenine dinucleotide; NAG, N‐acetyl‐β‐D‐glucosaminidase; NR, nicotinamide riboside; RBC, red blood cell; SD, standard deviation; T‐bilirubin, total bilirubin; WBC, white blood cell.

### 2.4 Secondary Endpoints: Effects on the Liver and Kidneys

AST levels (10.8 ± 23.5 U/L vs. −3.7 ± 19.3, *p* = 0.061) and alanine aminotransferase (ALT) levels (21.0 ± 39.7 U/L vs. 3.7 ± 28.3, *p* = 0.097) tended to increase from baseline following NR treatment compared with the placebo (Table 2, Figure 2). Despite these increases, no patients exhibited symptoms of liver dysfunction (Figure 2). Additionally, the observed elevations in AST and ALT were minor and remained well below levels typically seen in liver disease.

Estimated glomerular filtration rate (eGFR) calculated by serum creatinine (0.47 ± 7.4 mL/min/1.73 m^2^ vs. −10.7 ± 11.1, *p* = 0.088) tended to increase after NR treatment. Conversely, uric acid levels (0.18 ± 0.69 mg/dL vs. 0.80 ± 0.94, *p* = 0.088), Na (−1.83 ± 0.98 mEq/L vs. −0.83 ± 1.17, *p* = 0.076), and Cl (−1.67 ± 1.21 mEq/L vs. −0.83 ± 1.47, *p* = 0.093) tended to decrease after NR treatment. Therefore, NR may exert beneficial effects on the kidneys.

### 2.5 Secondary Endpoints: Effects on Atherosclerosis and Lipids

As a clinical evaluation of endothelial function and arterial stiffness, the cardio–ankle vascular index (CAVI) was used and was significantly improved after NR treatment (right: −0.4 ± 0.9 vs. 0.9 ± 0.8, *p* = 0.042*; left: −0.5 ± 0.9 vs. 0.7 ± 0.7, *p* = 0.046*) (Table 2, Figure 2). Conversely, the ankle–brachial index (ABI), an indicator of stenosis or occlusion of the lower extremity arteries, significantly decreased (worsened) after NR in the right leg (−0.1 ± 0.1 vs. 0.1 ± 0.1, *p* = 0.018*). The improvement in CAVI may have resulted from improvements in the arteries themselves, such as vessel stiffness and elasticity, independent of changes in blood pressure.

Serum lipoproteins were separated into 20 fractions using high‐performance liquid chromatography (HPLC). Cholesterol and triglyceride concentrations, as well as particle numbers per unit volume in each fraction, were measured using LipoSEARCH (Immuno‐Biological Laboratories Co. Ltd., Tokyo, Japan). The particle numbers per unit volume in the G15 and G16 fractions, which correspond to very large and large high‐density lipoprotein (HDL) particles, were significantly increased during the NR phase compared with the placebo phase (Tables S4 and S5). These findings suggest that NR may promote an increase in the size of large HDL particles. However, the sample size was small, and HDL‐C levels themselves did not show a significant rise.

### 2.6 Secondary Endpoints: Effects on Extremities Including Heel Pad Thickness and Skin Ulcers

Patients with WS often experience thin heel pads, skin ulcers, and pain due to heel pad thinning and ulceration. In this study, a reduction in heel pad thickness tended to be observed during the NR phase, but only in the left foot (left foot: 0.1 ± 1.6 mm vs. −0.7 ± 2.1, *p* = 0.060) (Table 2). The Numerical Rating Scale, a subjective pain assessment tool, showed no significant differences between the NR phase and the placebo phase.

Only three patients in this study had skin ulcers. However, ulcer size (horizontal diameter × vertical diameter) significantly decreased during the NR phase, whereas an increase was observed during the placebo phase (Table 3, Figure S1). The mean change from baseline in each of the 12 ulcers observed in the three patients was compared between the NR and placebo phases. In all three patients, NR was administered first, followed by a placebo. The mean ulcer size was reduced by −0.88 ± 1.64 cm^2^ during the NR phase, while it increased by 0.71 ± 1.02 cm^2^ during the placebo phase. Comparing the increase in the placebo phase with the reduction in the NR phase, there was a significant difference (*p* = 0.01*). These findings suggest that NR may ameliorate skin ulcers in patients with WS.

**TABLE 3:** Ulcer area identified at each visit.

	Baseline	After NR phase	After placebo phase
*n* (Missing)	10 (0)	6 (0)	9 (0)
Mean ± SD (cm^2^)	1.82 ± 1.31	1.26 ± 0.91	1.42 ± 0.79
Median (IQR) (cm^2^)	2.25 (0.25, 2.25)	1.25 (0.50, 2.25)	1.69 (1.00, 1.69)
Range (cm^2^)	0.25, 4.50	0.04, 2.25	0.25, 2.50
Mean changes of ulcer area compared with baseline ± SD (cm^2^)	NA	−0.88 ± 1.64	0.71 ± 1.02

*Note:* The ulcer area was calculated by multiplying the vertical and horizontal diameters of the ulcer.

Abbreviations: IQR, interquartile range; NA, not available; SD, standard deviation.

### 2.7 Secondary Endpoints: Effects on Visceral Fat Reduction

Accumulation of visceral fat relative to thin limbs, short stature, and low body weight is a characteristic feature of patients with WS. Although the difference between the NR phase and the placebo phase was not statistically significant, the visceral fat area at umbilical height, measured using computed tomography (CT), tended to decrease during the NR phase compared with the placebo phase (−18.3 ± 47.9 cm^2^ vs. −0.5 ± 48.4, *p* = 0.273) (Table 2).

### 2.8 Secondary Endpoints: Other Outcomes

No significant differences were observed between the NR and placebo phases in regard to changes from baseline regarding blood pressure; sarcopenia indicators, lipid metabolism parameters, glucose metabolism markers, and kidney function markers. Additionally, no significant changes were observed in blood cell counts or in other blood test parameters (Table 2). Individual patient data are presented in Tables S6–S11.

### 2.9 Exploratory Endpoint: Metabolome Analysis

**FIGURE 3:**
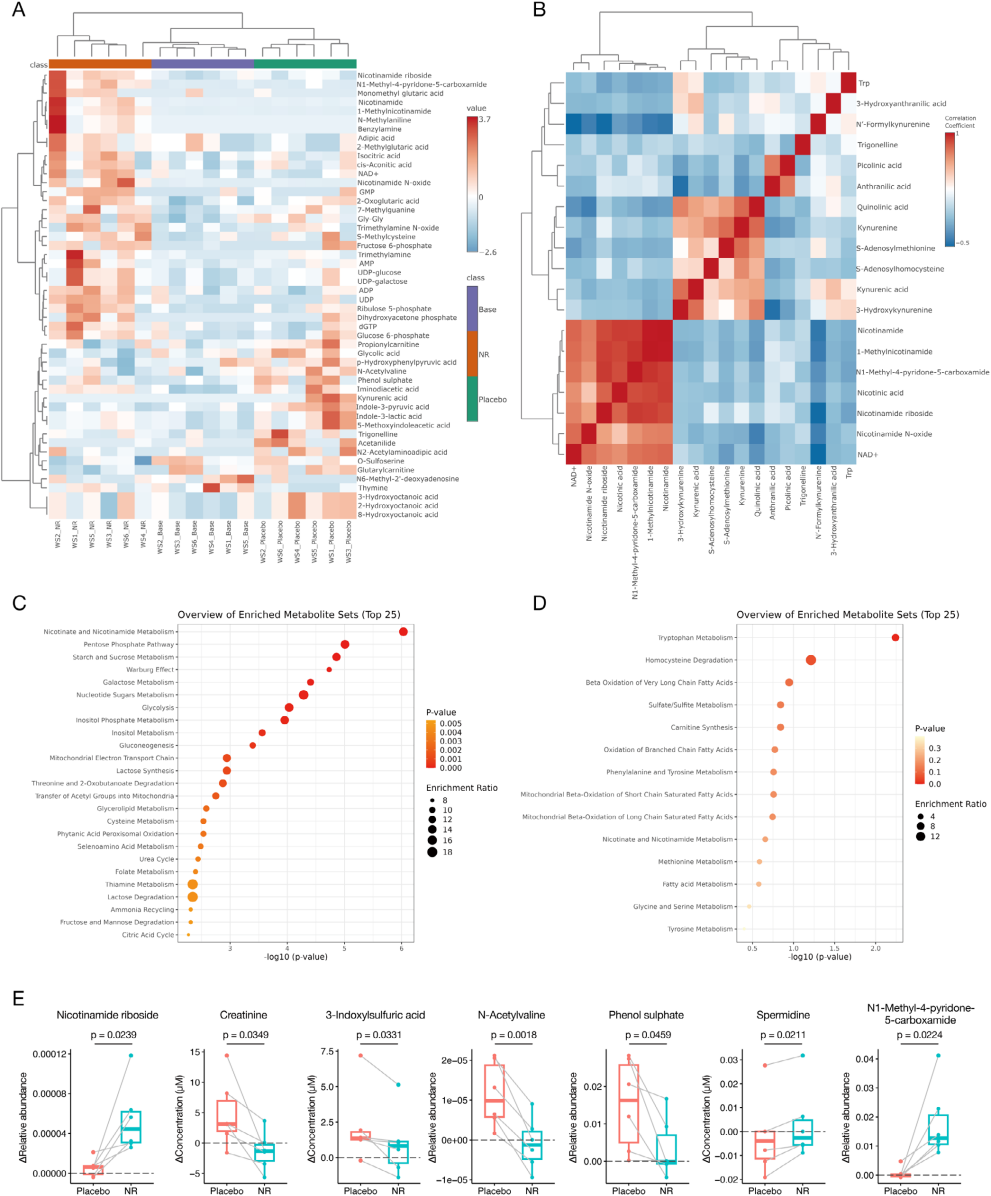
Results of metabolome analysis. (A) Hierarchical clustering analysis of the six patients using the top 50 metabolites with significant variations. (B) Correlation analysis of metabolites related to NAD^+^ synthetic pathways. (C) Enrichment analysis of significantly upregulated metabolites during NR treatment. (D) Enrichment analysis of significantly downregulated metabolites during NR treatment. (E) Metabolites with a significantly different expression between placebo and NR treatment. *P*‐values were calculated using a paired *t*‐test. Red bar and box show changes in the placebo phase, blue bar and box show changes in the NR treatment phase. Medium bars show the mean, top and bottom bars of the box show the standard deviation.

We performed a metabolome analysis on patient plasma as an exploratory study (Data S1). Hierarchical clustering analysis of the six participants who completed the trial is shown in Figure 3A. The analysis of the top 50 metabolites with significant variations revealed distinct changes in metabolite expression across the three groups: baseline, post‐NR, and post‐placebo. Focusing on metabolites related to NAD^+^, the results of the correlation analysis are shown in Figure 3B. The analysis suggested that although increases in NAD^+^, nicotinamide, and nicotinic acid were observed with NR treatment, the de novo pathway from tryptophan to NAD^+^ synthesis via 3‐hydroxykynurenine, 3‐hydroxyanthranilic acid, and quinolinic acid (Lautrup et al. 2024) was suppressed. Enrichment analysis also supported these findings, indicating that the Nicotinate and Nicotinamide Metabolism pathway was the top pathway among increased metabolites due to NR, whereas the Tryptophan Metabolism pathway was the top pathway among decreased metabolites (Figure 3C,D).

**FIGURE 4:**
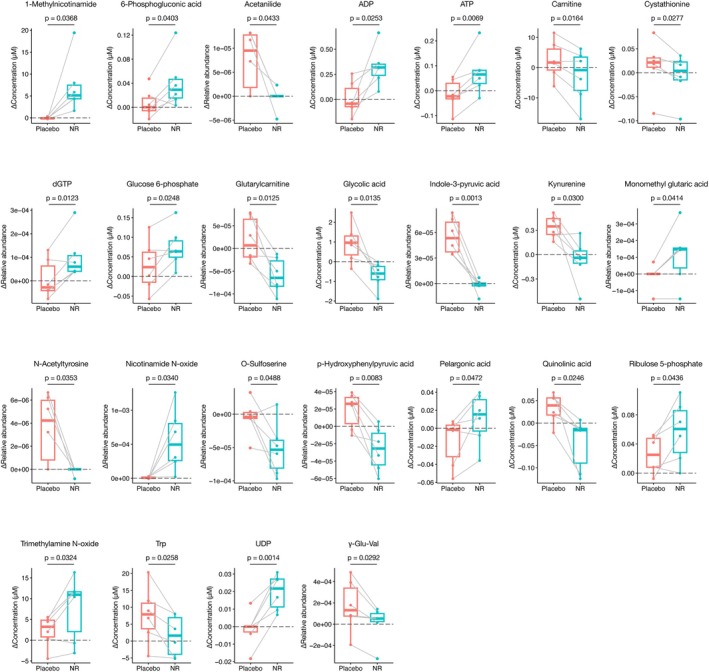
Metabolites with significantly different expression levels between the placebo and NR treatment groups. This figure displays metabolites with significantly different expression levels between the placebo and NR treatment groups. The *p‐values* were calculated using a paired *t*‐test. Red bars and boxes represent changes during the placebo phase, while blue bars and boxes represent changes during the NR phase. The mean is indicated by the middle bar, and the standard deviation is represented by the top and bottom bars of the box.

Figures 3E and 4 illustrate the individual metabolites with significant changes: 16 and 17 metabolites were up‐ and down‐regulated, respectively, during NR treatment. Notably, a decrease in creatinine in the NR administration group suggests a potential improvement in kidney function. Additionally, 3‐indoxylsulfuric acid, N‐acetylvaline, and phenol sulfate—metabolites associated with renal dysfunction and uremia (Kikuchi et al. 2019; Lee et al. 2022; Tanaka et al. 2015), decreased in the NR administration group, further indicating potential kidney function improvement. Moreover, the increase in spermidine, a compound with known anti‐aging properties (Madeo et al. 2018), was observed in the NR administration group. Conversely, N1‐Methyl‐4‐pyridone‐5‐carboxamide (4PY), which could potentially contribute to major adverse cardiovascular events (MACE) (Ferrell et al. 2024), increased with NR treatment, consistent with previous reports (Yoshino et al. 2021).

## 3 Discussion

This study is the first to demonstrate that NR administration increases plasma NAD^+^ levels in patients with WS. Although AST and ALT levels tended to increase slightly, no adverse events were reported during the NR phase. Importantly, CAVI was significantly improved, and skin ulcer areas were reduced. These findings suggest that NR administration may help improve atherosclerosis and skin ulcers—two key features of WS. Furthermore, metabolome analysis indicated a significant decrease in plasma creatinine, suggesting potential improvement in renal function.

### 3.1 NR Effects on the Liver

Previous studies have demonstrated improvements in NAFLD with NR administration in mice (Han et al. 2019), and reductions in hepatic lipid content in patients with obesity following NR supplementation (Dollerup et al. 2018). Furthermore, ALT and gamma‐glutamyl transferase levels were shown to decrease in patients with NAFLD treated with a combination of nicotinamide riboside and pterostilbene (NRPT) (Dellinger et al. 2023). Given the high prevalence of fatty liver complications in WS patients (Koshizaka et al. 2020), we anticipated an improvement in liver function with NR administration. However, in our study, AST and ALT levels tended to increase during NR administration compared with the placebo phase.

Several factors may explain this unexpected outcome. Patients with WS inherently have elevated AST and ALT levels and a higher prevalence of fatty liver, making them more sensitive to changes in liver function. Since NR is rapidly absorbed and transiently metabolized by the liver, it may cause a temporary increase in NAD^+^ levels. Additionally, patients with WS frequently experience skin ulcers and wound infections, often requiring antibiotics and nonsteroidal anti‐inflammatory drugs, which can exacerbate liver function. Therefore, careful consideration is necessary when administering NR in this population.

Notably, patients who exhibited increased AST and ALT levels during NR treatment were all female (Tables S6–S8). Given that male and female patients in this study had similar body sizes, this suggests that sex‐based differences in pharmacokinetics or hormonal influences, rather than body size, may affect NR metabolism. Therefore, sex‐specific dosing strategies should be considered, with potential dose reductions for female patients with WS receiving NR treatment.

### 3.2 NR Effects on the Kidney

Although eGFR declines with age, the rate of decline is more rapid in patients with WS (Maeda et al. 2023). In this study, the rate of eGFR decline, as calculated using serum creatinine, tended to slow during NR treatment. Additionally, plasma creatinine levels measured via metabolomic analysis were significantly lower after NR administration. These findings suggest that NR may have helped suppress renal functional decline. Furthermore, when considered alongside improvements in CAVI and skin ulcers, these results suggest that enhanced renal blood flow, potentially due to reduced arterial stiffness, may have contributed to the observed benefits.

### 3.3 NR and Malignancy

One patient who exhibited renal enlargement at the end of the NR treatment phase was found to have a renal mass at the end of the placebo phase (Tables S7 and S8). This patient subsequently underwent examination and treatment. Notably, no tumors were present at enrollment; otherwise, the patient would have been excluded from the study. In patients with WS, malignant tumors tend to develop at a relatively young age (Onishi et al. 2012; Maeda et al. 2023).

There has been some discussion about whether NR supplementation might promote malignancies due to the upregulation of NAD^+^ metabolic enzymes. This remains an important consideration, and for this reason, patients with overt cancers were not included in this study. However, some research suggests that NR may have anticancer properties (Feng et al. 2024; Tummala et al. 2014; Santidrian et al. 2013; Ren et al. 2017; Pang et al. 2023). Given the uncertainty surrounding NR's effects on tumorigenesis, NAD^+^ supplementation should likely be avoided in patients with known malignancies until further evidence clarifies its role.

### 3.4 NR Effects on Atherosclerosis

Recent human intervention studies on NAD^+^ supplementation, including NR, have demonstrated improvements in glucose tolerance and insulin resistance, reductions in blood pressure, and enhanced carotid–femoral pulse wave velocity (PWV) (Martens et al. 2018; Yoshino et al. 2021). In patients with peripheral artery disease, 6 months of NR administration significantly improved the 6‐min walking distance (McDermott et al. 2024). Similarly, our study showed an improvement in CAVI during the NR phase.

CAVI is a noninvasive indicator of arterial stiffness developed in Japan. It provides a reliable measure of arterial stiffness that is independent of blood pressure (Shirai et al. 2006).

Metabolome analysis revealed that methylated arginine derivatives, including asymmetric dimethyl arginine (ADMA) and L‐N monomethyl arginine (L‐NMMA, Nω‐Methylarginine), tended to decrease during NR treatment (Figure S2). Elevated levels of ADMA and L‐NMMA directly or indirectly reduce nitric oxide synthesis (Liu et al. 2018; Víteček et al. 2012). Therefore, their reduction may have contributed to the observed improvement in vascular endothelial function and CAVI.

Higher CAVI values have been significantly correlated with the presence of atherosclerotic lesions (Park et al. 2013). Several prospective studies have also identified high CAVI as a risk factor for cardiovascular events (Sato et al. 2016; Satoh‐Asahara et al. 2015). Therefore, a reduction in CAVI following NR administration may lower the risk of cardiovascular disease events.

The ABI showed a downward trend after the NR phase, suggesting a potential worsening. However, while ABI is useful for assessing the need for surgical intervention, its sensitivity in detecting minor hemodynamic changes during medical therapy is limited. Clinically, ABI changes of < 0.15 are generally considered insignificant. Therefore, the ABI variations observed in this study are unlikely to be of clinical relevance. Further validation studies with larger patient populations are needed.

### 3.5 Effects of NR on Extremities, Heel Pad Thickness, and Skin Ulcers

During NR treatment, skin ulceration decreased in size, and heel pad thinning tended to be prevented on one side. Patients with WS exhibit abnormal connective tissue metabolism, characterized by subcutaneous tissue atrophy, reduced blood flow, and decreased fibroblast activity. These factors contribute to the development of intractable skin ulcers, a hallmark of WS (Kinoshita et al. 2023; Motegi et al. 2021). Such ulcers are painful and significantly reduce the quality of life (Kitagawa et al. 2023).

NR administration has been shown to improve mitochondrial function and may enhance fibroblast activity, thereby preventing skin and subcutaneous tissue atrophy (Sun et al. 2020). Additionally, NAD^+^ supplementation has been reported to blunt Th17 inflammation via arginine biosynthesis and redox control in patients with psoriasis (Han et al. 2023). These anti‐inflammatory effects, combined with enhanced arginine biosynthesis, may contribute to ulcer healing.

NR supplementation has also been shown to promote wound healing in diabetic rat models by modulating autophagy and apoptosis (Siri et al. 2025). This suggests that NR could aid in ulcer treatment by addressing unfolded protein response dysregulation and pyroptosis. However, given that only three patients in this study had ulcers, the sample size is too small to draw definitive conclusions. Further investigation with a larger cohort is necessary to fully assess the effects of NR on skin ulcer healing.

### 3.6 Effects of NR on Visceral Fat

During the NR phase, some patients exhibited a tendency toward decreased visceral fat areas. Since patients with WS typically accumulate visceral fat, which contributes to insulin resistance, reducing visceral fat may provide metabolic benefits. A clinical study previously reported that NR supplementation improved body composition in individuals with obesity (Remie et al. 2020). Additionally, NR treatment in WRN‐knockout adipocytes has been shown to normalize the expression of adipocyte‐related genes (Tian et al. 2024), which may underlie the observed trend of visceral fat reduction in this study.

Furthermore, free fatty acids released from visceral fat may enter the liver, potentially leading to elevated liver enzyme levels while simultaneously reducing visceral fat stores. Taken together, these findings suggest that NR supplementation may help mitigate visceral fat accumulation in patients with WS.

### 3.7 Metabolome Analysis

A previous metabolome analysis showed that metabolites such as 3‐indoxylsulfuric acid, N‐acetylglucosamine, and phenol sulfate, which accumulate due to renal dysfunction and uremia (Kikuchi et al. 2019; Lee et al. 2022; Tanaka et al. 2015), were reduced after NR administration. 3‐indoxylsulfuric acid is known as a uremic substance, and its association with sarcopenia has been suggested in previous reports (Lee et al. 2022). Phenol sulfate has attracted attention as a causative agent of diabetic nephropathy (Kikuchi et al. 2019). Considering that sarcopenia is almost always present in WS cases (Yamaga et al. 2017b) and that renal dysfunction is severe as mentioned above (Maeda et al. 2023), these substances may be contributing to the disease phenotype, and NR may lead to symptom suppression.

### 3.8 HyperPARylation

HyperPARylation refers to excessive poly(ADP‐ribosyl)ation (PARylation), a post‐translational modification primarily regulated by poly(ADP‐ribose) polymerase (PARP). PARylation plays a critical role in DNA damage response and cellular stress regulation. PARP detects DNA damage and utilizes NAD^+^ as a substrate to facilitate DNA repair. In disease states characterized by excessive DNA damage, PARP becomes hyperactivated, resulting in abnormally high levels of PARylation. This excessive activation leads to massive NAD^+^ consumption (Sriram et al. 2016; Ray Chaudhuri and Nussenzweig 2017). NAD^+^ is essential for cellular energy metabolism (ATP production) and DNA repair. Its depletion results in mitochondrial dysfunction, contributing to aging and disease progression. HyperPARylation and reduced NAD^+^ levels have been observed in rare diseases associated with impaired DNA repair, including WS (Fang et al. 2019), Cockayne syndrome (Scheibye‐Knudsen et al. 2014), ataxia telangiectasia mutated (ATM) deficiency (Fang et al. 2016), as well as neurodegenerative diseases such as Alzheimer's and Parkinson's disease (Lautrup et al. 2024).

### 3.9 Prospects

The positive finding that NR intervention increases the levels of NR and NAD+ in the blood of patients with WS may extend to other diseases in this category with hyperPARylation. Indeed, a recent clinical intervention in Ataxia telangiectasia demonstrated the benefits of NAD^+^ supplementation (Presterud et al. 2024).

NAD^+^ levels were evaluated using blood samples; however, NAD^+^ levels in tissues might show greater differences. Unfortunately, we currently lack the techniques to measure NAD^+^ in tissues. Since higher doses of NR are now considered safe, future studies using larger doses may yield even better results.

### 3.10 Limitations

This study had some limitations. First, the number of patients was small because this was a single‐center study, which coincided with the COVID‐19 pandemic. Increasing the number of enrolled patients would enhance the statistical power and informativeness of the findings. Nevertheless, this is the first double‐blind, placebo‐controlled trial to demonstrate the safety of NR and its beneficial effects on CAVI, skin ulcers, and creatinine levels analyzed via metabolomics in patients with WS. Second, some patients in the placebo group discontinued treatment due to aspiration pneumonia or malignancy, both of which are potential complications of WS. Third, this study did not include a washout period. While NR has been shown to elevate NAD^+^ levels in human blood within hours (Trammell et al. 2016b), a previous crossover study reported an anti‐inflammatory effect of NR following a 21‐day administration period with a subsequent 2–3 week washout phase (Elhassan et al. 2019). However, basic research has indicated that NR effects dissipate rapidly after discontinuation (Marmolejo‐Garza et al. 2024; Chu et al. 2022). Given that NR was administered for 28 weeks in this study, any potential carryover effects are likely to be minimal.

## 4 Conclusion

NR treatment for patients with WS showed a significant improvement in arterial stiffness through both CAVI and the reduction of skin ulcer area without the occurrence of serious adverse events. Additionally, metabolome analysis indicated that NR slowed renal dysfunction. These findings suggest that NR may be beneficial for preventing atherosclerosis, skin ulcers, and kidney impairment in patients with WS.

## 5 Methods

### 5.1 Study Overview

This was a phase 1 and 2 double‐blind randomized crossover, placebo‐controlled study. The study protocol and informed consent forms were approved by the Certified Clinical Research Review Board of Chiba University Hospital. The ethics approval number is CRB3180015. This study was conducted in compliance with the principles of the Declaration of Helsinki. All analyses were performed by Chiba University, independent of the sponsor, according to a prespecified statistical analysis plan. This study was registered with the Japan Registry of Clinical Trials (jRCTs031190141).

### 5.2 Patients

Patients were recruited between March 1, 2021, and September 30, 2022, and all enrolled patients provided written informed consent. Patients who met the following criteria were included in this study: patients who were diagnosed with WS based on the criteria for WS (Takemoto et al. 2013) and those who met the inclusion criteria without violating the exclusion criteria (Table S12).

As a rationale for selecting patients, WS is a rare disease, and there are approximately 2000 patients with this disease in Japan. Therefore, the policy was to register patients with diseases who met the eligibility criteria and did not violate the exclusion criteria, which were patients with advanced diseases that required supportive care, abnormal liver function, or malignant tumors. The sample size for this study was determined since WS is a rare disease and the patients visiting our hospital are approximately 16. To increase the reliability of the study results, the sample size was expanded to 30 patients to recruit patients from all over the country, as there were approximately 40 patients registered in the Japanese WS Registry.

### 5.3 Study Design

Eligible patients were enrolled by facsimile through the Chiba University Clinical Trial Data Center. To reduce variability among participants, registration was stratified by sex as an allocation factor. Participants were randomly assigned to either the NR pretreatment group or the placebo pretreatment group in a 1:1 allocation. A minimization method was applied using the data management system DATATRAK (NTT Data, Tokyo, Japan), located at the registration center. This system was concealed from both researchers and participants. Only two physicians, who handled the study drugs but did not meet the participants, were aware of the allocation.

**FIGURE 5:**
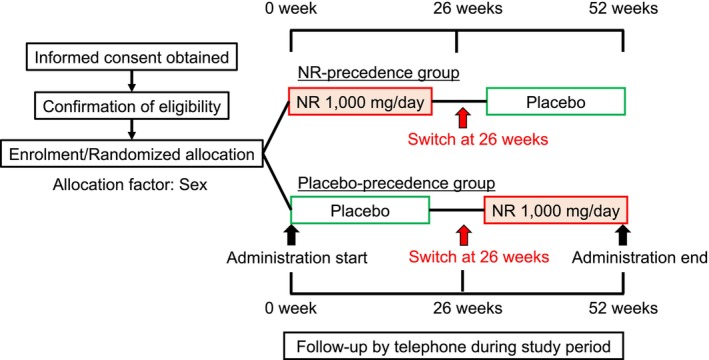
A flow chart showing the study design. NR, nicotinamide riboside.

NR and the placebo were provided by ChromaDex (Los Angeles, CA, USA). The drugs were self‐administered once daily for 26 weeks. Patients took oral capsules containing 1000 mg of NR or placebo capsules containing microcrystalline cellulose, with both capsules being visually identical to ensure blinding. After 26 weeks, the patients crossed over to the other study arm (Figure 5). A washout period was not implemented since the half‐life of NR is short.

Patients were initially assessed based on their current medical records. After a preliminary determination of eligibility, patients were screened again (visit 1) at the Division of Diabetes, Metabolism, and Endocrinology at Chiba University Hospital. If eligibility was confirmed, patients were enrolled in the study, and initial laboratory and clinical data were collected. The study drug or placebo was then prescribed. Follow‐up phone calls were made at 4, 8, 12, 16, and 20 weeks to monitor adherence and adverse events.

After 26 weeks, patients returned for a second examination (visit 2). Subsequently, they were crossed over to the opposite arm of the study, and either the placebo or NR was administered for an additional 26 weeks. Follow‐up telephone calls were made at 30, 34, 38, 42, and 46 weeks after visit 1 to ensure adherence to study requirements and to monitor adverse events. The patients visited the hospital at 52 weeks for a third examination (visit 3).

The primary (safety) endpoint was the evaluation of NR safety at 26 and 52 weeks in patients with WS. The incidence, frequency, and severity of adverse events following NR or placebo administration were evaluated. All adverse events, whether causally related to the study drugs or not, were monitored until resolution or for 4 weeks after study completion or discontinuation. All adverse events judged to be causally related to the study drugs and severity by physicians were also reported as the same. The number of events and the frequency and condition of occurrence were also reported. All adverse events recorded during the study period were coded according to the Medical Dictionary for Regulatory Activities (MedDRA) version 24.1. The severity of adverse events was classified as mild (only clinical or laboratory findings without subjective symptoms), moderate (requiring some treatment), and severe (leaving significant impairment or life‐threatening).

Secondary endpoints evaluated efficacy in terms of changes in: skin ulcer number and size, amount of change of NAD^+^ levels measured by metabolome analysis, inflammatory response (CRP), blood tests (lipid, glucose, liver, renal functions, blood cell counts, total protein, albumin, uric acid, CPK, Na, K, Cl), eGFR calculated by serum creatinine levels using the Cockroft–Gault method, urine albumin excretion, urine NAG, beta‐2‐microglobulin, liver‐type fatty acid‐binding protein (L‐FABP), kidney injury molecule‐1 (Kim‐1), handgrip strength, walking speed, visceral fat area, CAVI, ABI, skeletal muscle index by dual‐energy X‐ray absorptiometry (DEXA), and heel pad thickness measured by X‐ray.

The examinations used fasting blood and urine samples, ensuring accuracy and reproducibility through standardized protocols. Grip strength was measured in the standing position whenever possible, once on each side. Walking speed was evaluated using a 10‐m walking test. The visceral fat area was measured by CT by radiologists blinded to treatment assignment. CAVI and ABI were measured using the VaSera VS‐3000 vascular screening system (Fukuda Denshi, Tokyo, Japan). Additionally, metabolome analysis was performed as an exploratory endpoint.

### 5.4 Analysis of the Number of Lipoprotein Particles in Lipoprotein Fractions

Serum lipoproteins were fractionated into 20 fractions by HPLC, and cholesterol and triglyceride concentrations in each fraction were measured using LipoSEARCH. Lipoprotein particle number per unit volume in each fraction was calculated using an algorithm developed by Skylight Biotech (Tokyo, Japan).

A detailed algorithm for determining the lipoprotein particle numbers per unit volume is available in the International Patent Register (https://patentscope.wipo.int/search/; patent number: WO2015/152371). A Mann–Whitney U test was performed to compare lipoprotein particle numbers per unit volume in each fraction before and after NR and placebo administration.

### 5.5 Metabolome Analysis

Plasma metabolome analysis of samples was conducted by Human Metabolome Technologies Inc. (HMT), (Tsuruoka, Yamagata, Japan) according to HMT's ω Scan package, using capillary electrophoresis Fourier transform mass spectrometry (CE‐FTMS) based on the methods described previously (Sasaki et al. 2019). Data were analyzed using MetaboAnalyst 6.0 (Pang et al. 2024). Missing data were imputed using the half‐minimum method.

### 5.6 Statistical Analysis of Primary and Secondary Endpoints

The distribution and summary statistics of patient background data in each analyzed population were calculated for each group. Continuous variables were presented as summary statistics (number of patients, mean, standard deviation (SD), median, and interquartile range (IQR)) and were calculated for each group, whereas categorical variables were described as frequencies and proportions.

Safety and efficacy analyses were also performed. To analyze the primary endpoint and safety of NR in patients with WS at 26 and 52 weeks, the incidence, frequency, and severity of adverse events occurring after NR administration were evaluated using a safety analysis set (SAF). The MedDRA version 24.1 was used to describe adverse events. To analyze the secondary endpoint, the efficacy of the following items was evaluated using a full analysis set (FAS). The per‐protocol set (PPS) was equivalent to the FAS. The size and number of skin ulcers were tabulated at the baseline (day 0), 26‐week, and 52‐week periods. For the remaining items, the amount and percentage of change were calculated from the values at the baseline (day 0), 26‐week, and 52‐week periods. A paired two‐tailed t‐test was used for comparison between groups. For each variable, patients with missing values during either phase were excluded from the analysis. All statistical analyses were performed by statisticians based on a prespecified statistical analysis plan, using SAS (version 9.4; SAS Institute, Cary, NC, USA), JMP (version 15.0; SAS, Cary, NC, USA), or R software (V.3.2.0; https://cran.r‐project.org/; R Foundation for Statistical Computing, Vienna, Austria).

## Author Contributions

All the authors made significant contributions to this study. Masaya Koshizaka and Koutaro Yokote conceptualized the design. Mayumi Shoji, Hisaya Kato, and Masaya Koshizaka wrote the manuscript – original draft. Masaya Koshizaka and Mayumi Shoji managed the project. Hisaya Kato, Yusuke Baba, Vilhelm A. Bohr, and Masaya Koshizaka reviewed and edited the manuscript. Hiyori Kaneko, Takahiro Ishikawa, Naoya Teramoto, Daisuke Kinoshita, Ayano Yamaguchi, Yukari Maeda, Yoshiro Maezawa, and Koutaro Yokote reviewed the manuscript. Masaya Koshizaka and Yoshiro Maezawa designed the protocol. Mayumi Shoji, Masaya Koshizaka, Hisaya Kato, and Yoshiro Maezawa recruited patients and conducted physical examinations, including blood sample collection. Hiyori Kaneko and Takahiro Ishikawa administered the study drugs. Yosuke Inaba, Yuki Shiko, and Yoshihito Ozawa performed statistical analyses of primary and secondary endpoints. Hisaya Kato and Masaya Koshizaka conducted metabolome analyses. Vilhelm A. Bohr supervised the study. All the authors have read the final manuscript and approved its publication. Koutaro Yokote is the guarantor of this work, has full access to all the study data, and takes responsibility for the integrity and accuracy of the data and the analysis.

## Disclosure

Some figures in the graphical abstract were used with permission from Sanyo Media Co. Ltd.

## Ethics Statement

This was a Phase 1 and 2 double‐blind, randomized, crossover, placebo‐controlled study. The study protocol and informed consent forms were approved by the Certified Clinical Research Review Board of Chiba University Hospital (ethics approval number: CRB3180015). The study was conducted in compliance with the principles of the Declaration of Helsinki. All analyses were performed by Chiba University, independent of the sponsor, according to a prespecified statistical analysis plan. The study was registered with the Japan Registry of Clinical Trials (jRCTs031190141).

## Conflicts of Interest

The authors declare no competing interests. ChromaDex (Los Angeles, CA, USA) provided the study drugs, NR, and placebo. Chiba University and ChromaDex signed contracts to conduct this study. ChromaDex was not involved in the study design, execution, data analysis, and interpretation, manuscript preparation, or the decision to publish. The funding source was not involved in the study design, or the collection, analysis, and interpretation of data, writing of the report, and the decision to submit the article for publication.

## Supporting information


**Figure S1.** Ulcer area of a patient at baseline, NR phase, and placebo phase, This figure shows the ulcer area of a patient at each visit. The ulcer is located on the right Achilles tendon and is marked with red circles in the lower photos. After the NR phase, epithelial formation is evident.

**Figure S2.** Metabolites related to nitric oxide synthesis. This figure presents metabolites related to nitric oxide synthesis. The *p‐*values were calculated using a paired *t*‐test. Red bars and boxes represent changes during the placebo phase, whereas blue bars and boxes represent changes during the NR phase. The mean value is indicated by the middle bar, and the standard deviation is represented by the top and bottom bars of the box.

**Table S1.** Clinical trials of nicotinamide riboside.
**Table S2.** Clinical studies of NAD^+^ intermediate metabolites.
**Table S3.** Adverse events were observed in the patients.
**Table S4.** Serum lipoprotein particle number per unit volume in each fraction at baseline was measured using high‐performance liquid chromatography.
**Table S5.** High‐performance liquid chromatography measurements of changes in serum lipoprotein particle number per unit volume across each fraction compared to baseline.
**Table S6.** Individual patient characteristics at baseline.
**Table S7.** Changes in clinical parameters after NR phase from baseline.
**Table S8.** Changes in clinical parameters after the placebo phase from baseline.
**Table S9.** Serum lipoprotein particle number per unit volume in each fraction was measured using high‐performance liquid chromatography at baseline.
**Table S10.** Changes of serum lipoprotein particle number per unit volume in each fraction compared to baseline measured using high‐performance liquid chromatography after NR phase.
**Table S11.** Changes of serum lipoprotein particle number per unit volume in each fraction compared to baseline measured using high‐performance liquid chromatography after the placebo phase.
**Table S12.** Inclusion and exclusion criteria.

**Data S1.** Metabolome data from plasma samples of six patients at baseline, post‐NR administration, and post‐placebo administration.

## Data Availability

Data related to the primary results, as presented in the paper, are available under restricted access as ethical approval is required as additional processing or analysis by third parties not involved in the clinical study was not covered in the approved protocol or patient consent. Access can be obtained, contingent on appropriate ethics approval and data sharing agreements, by contacting MK (overslope@chiba-u.jp) for the purposes of confirming the analysis in the paper. Responses to valid requests will be reasonably provided within 10 working days of receipt. This data‐sharing process will be available starting 1 year and ending 5 years after the publication of this article. However, raw individual‐level participant data are not available due to Japanese regulations and the terms of the informed consent form, which do not permit sharing such data with third parties beyond the scope of the original study.
