# Supplementary figures and images for: Nicotinamide Riboside Supplementation Benefits in Patients With Werner Syndrome: A Double‐Blind Randomized Crossover Placebo‐Controlled Trial
Source: Aging Cell. 2025 Jun 3;24(8):e70093. doi: 10.1111/acel.70093 (PMC12341770; doi:10.1111/acel.70093)

Supplementary Figure 1

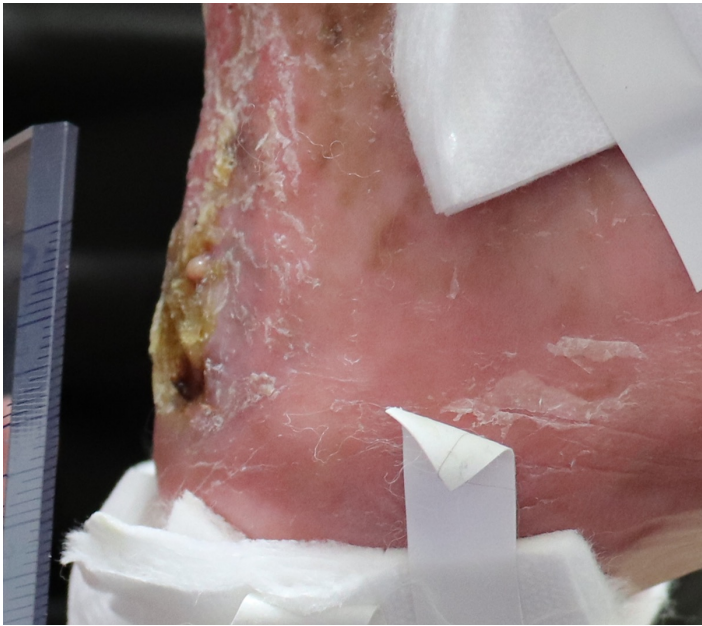

Baseline

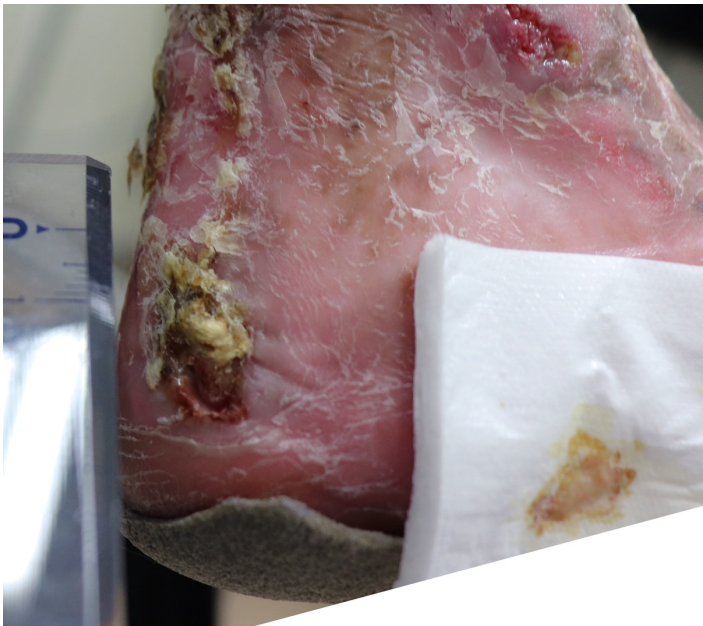

After NR phase

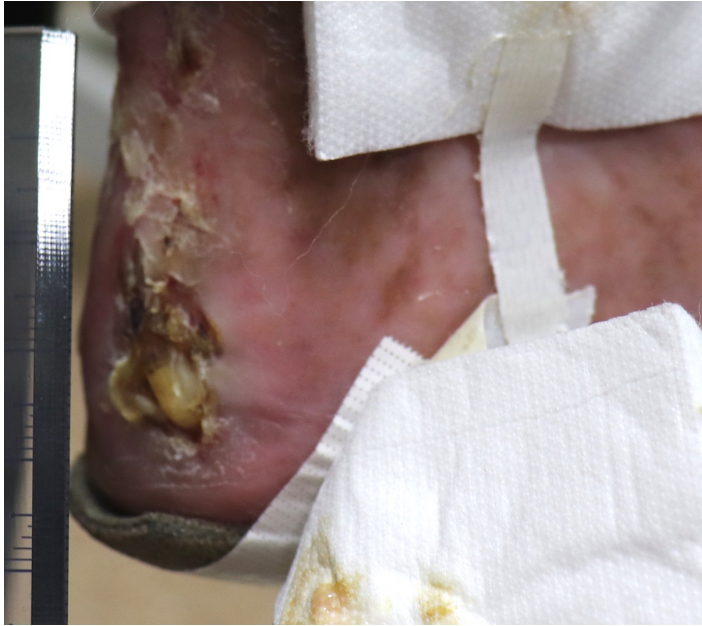

After placebo phase

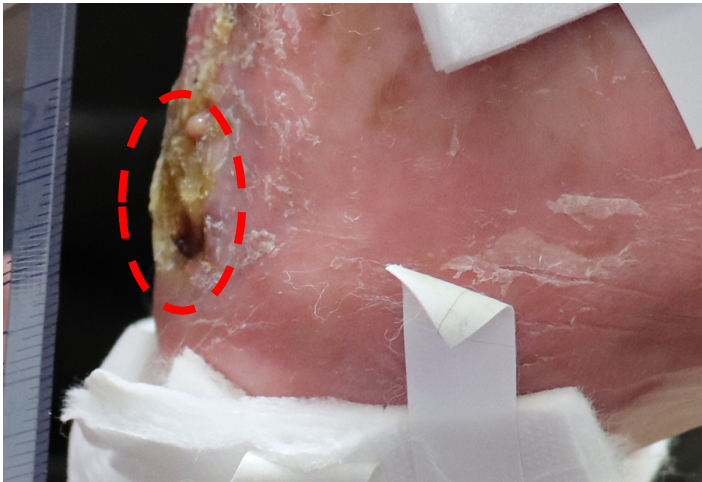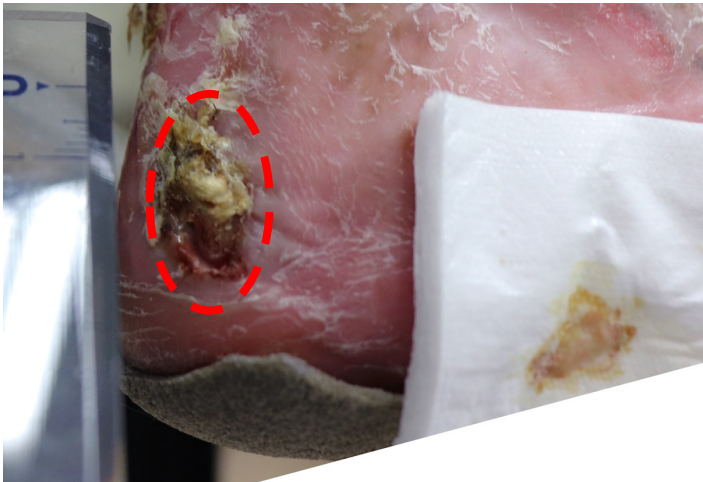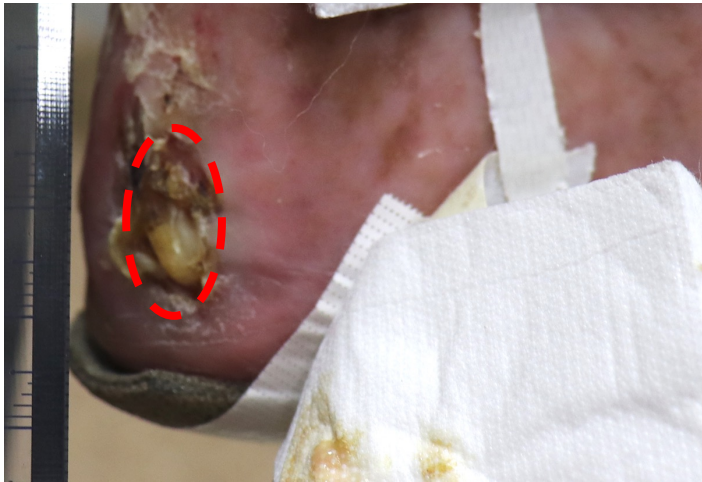

Supplement: Supplementary file 1 — Figure S1. Ulcer area of a patient at baseline, NR phase, and placebo phase, This figure shows the ulcer area of a patient at each visit. The ulcer is located on the right Achilles tendon and is marked with red circles in the lower photos. After the NR phase, epithelial formation is evident. [file ACEL-24-e70093-s003.pdf]

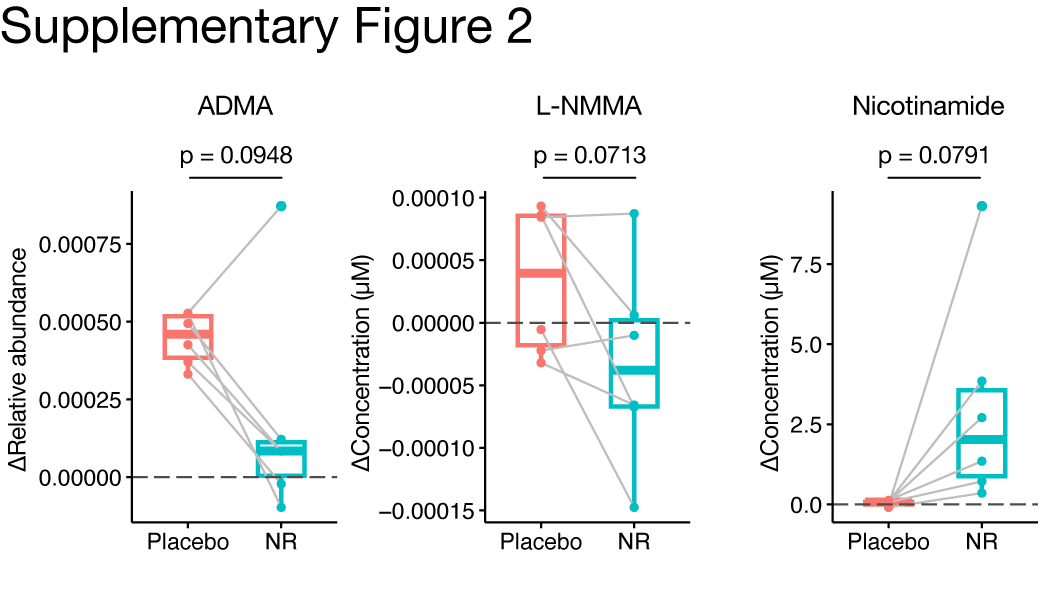

Supplement: Supplementary file 2 — Figure S2. Metabolites related to nitric oxide synthesis. This figure presents metabolites related to nitric oxide synthesis. The p‐values were calculated using a paired t‐test. Red bars and boxes represent changes during the placebo phase, whereas blue bars and boxes represent changes during the NR phase. The mean value is indicated by the middle bar, and the standard deviation is represented by the top and bottom bars of the box. [file ACEL-24-e70093-s004.tif]
