# Supplementary material for: Nicotinamide Riboside Supplementation Benefits in Patients With Werner Syndrome: A Double‐Blind Randomized Crossover Placebo‐Controlled Trial
Source: Aging Cell. 2025 Jun 3;24(8):e70093. doi: 10.1111/acel.70093 (PMC12341770; doi:10.1111/acel.70093)
Supplement: Supplementary file 3 — Table S1. Clinical trials of nicotinamide riboside. Table S2. Clinical studies of NAD+ intermediate metabolites. Table S3. Adverse events were observed in the patients. Table S4. Serum lipoprotein particle number per unit volume in each fraction at baseline was measured using high‐performance liquid chromatography. Table S5. High‐performance liquid chromatography measurements of changes in serum lipoprotein particle number per unit volume across each fraction compared to baseline. Table S6. Individual patient characteristics at baseline. Table S7. Changes in clinical parameters after NR phase from baseline. Table S8. Changes in clinical parameters after the placebo phase from baseline. Table S9. Serum lipoprotein particle number per unit volume in each fraction was measured using high‐performance liquid chromatography at baseline. Table S10. Changes of serum lipoprotein particle number per unit volume in each fraction compared to baseline measured using high‐performance liquid chromatography after NR phase. Table S11. Changes of serum lipoprotein particle number per unit volume in each fraction compared to baseline measured using high‐performance liquid chromatography after the placebo phase. Table S12. Inclusion and exclusion criteria. [file ACEL-24-e70093-s002.docx]

**Supplementary Table 1. Clinical trials of nicotinamide riboside**

| Participants | Dose  (mg/day) | Study duration | Study design | Sample size | Outcomes or Results | Evaluation method | NAD⁺ metabolism | Supplementary References |
| --- | --- | --- | --- | --- | --- | --- | --- | --- |
| Healthy volunteers | 1000 | 7 days | Pilot study | 1 healthy volunteer | NR pharmacokinetics | Blood | PBMNs NAD⁺ elevated | 1 |
|  | 100/ 300/ 1000 | Single administration | Randomized, double-blind, 3-arms crossover | 12 healthy volunteers | NR pharmacokinetics, No subjective symptoms | Blood, Subjective symptoms | PBMNs NAD⁺ elevated | 1 |
|  | 250-2000 | 9 days | Non-randomized, open label | 8 healthy volunteers | NR pharmacokinetics, No side effects | Blood, Vital | NR and NAD⁺ in whole blood elevated | 2 |
|  | 100/ 300/ 1000 | 8 weeks | Randomized, double-blind, placebo-controlled | 35 healthy volunteers / group | NR pharmacokinetics, No side effects | Blood, Vital | NAD⁺ in whole blood, NAM metabolites in urine elevated | 3 |
|  | 500 | Single | Randomized, double-blind, placebo-controlled, crossover | Young adults (22.9±1.0 yrs)  Old adults (71.5±1.0 yrs)  12 persons / group | Maximal muscle strength in the old adults, improvement of fatigue | Blood, Muscle test | NAM metabolites in urine elevated | 4 |
|  |  |  |  |  | Hepatic fat mass reduction tendency, No change in insulin resistance, energy metabolism, and body composition | MR, DXA spectroscopy, HECP, indirect calorimetry, | NAM metabolites in urine elevated | 5 |
|  | 1000 | 6 weeks | Randomized, double-blind, placebo-controlled, crossover | 12 healthy volunteer / group | Blood pressure lowering, cfPWV improvement, no change in glucose and energy metabolism, muscle strength and walking distance, and body composition | PWV, IVGTT, HOMA, indirect calorimetry, walking load and grip strength test, DXA | PBMNs NAD⁺  elevated | 6 |
| Patients | 2000 | 12 weeks | Randomized, double-blind, placebo-controlled | 20 obese male / group | No change in glucose tolerance, insulin secretion, and incretin secretion | 75gOGTT |  | 7 |
|  |  |  |  |  | No change in NAD⁺ amount in lateral vastus muscle, mitochondrial function and amount in skeletal muscle, and skeletal muscle fat mass | Lateral vastus muscle biopsy, |  | 8 |
|  |  |  |  |  |  | 1H-MR spectroscopy |  |  |
|  | 1000 | 6 weeks | Randomized, double-blind, placebo-controlled, crossover | 13 obese male and female | Fat mass decrease and lean body mass increase, energy metabolism during sleeping increase, accumulation of acetylcarnitine, Plasma inflammatory markers decreasing trend  No change in skeletal muscle mitochondrial function, insulin resistance, myocardial ejection fraction, blood pressure | H-MR spectroscopy indirect calorimetry, lateral vastus muscle biopsy, High-resolution respirometry, air displacement method | Skeletal muscle NAD^+^ synthesis system, NAM metabolites elevated | 9 |
|  | 1000 | 21 days | Randomized, double-blind, placebo-controlled, crossover | 12 old healthy men (70–80 years old) | Decreased inflammatory cytokines, | Lateral vastus muscle biopsy, High-resolution respitometry,75gOGTT | Skeletal muscle NAD⁺ synthesis system, NAM metabolites, NAD^+^ in whole blood elevated | 10 |
|  |  |  |  |  | No change in skeletal muscle mitochondrial amount and function, grip strength, glucose tolerance, and insulin resistance |  |  |  |
|  | 1000 | 30 days | Double-blind phase I clinical trial. | 30 patients with Parkinson’s disease. | Increase in cerebral NAD levels, brain NAD levels exhibited altered cerebral metabolism, NR decreased the levels of inflammatory cytokines in serum and cerebrospinal fluid | 31phosphorous magnetic resonance spectroscopy | NR augmented the NAD metabolome in blood cells and/or skeletal muscle. | 11 |
|  | 1000  +200mgPT | 30 days | double-blind, placebo-controlled study | 32 old healthy men (55–80 yrs) | Not improve recruitment of the MuSC pool or other measures of muscle recovery in response to injury or subsequent regeneration | The MuSC pool |  | 12 |
|  | 1000 | 6 weeks | randomized, placebo-controlled, double-blind, crossover trial | 25 patients with an estimated glomerular filtration rate (eGFR) of less than 60mL/min/1.73 m^2^. | NR did not improve VO2 peak or total work efficiency. | Exercise tolerance and metabolic profile | Markers of systemic mitochondrial metabolism and lipid profiles improved. | 13 |
|  | NRPT1g/ NRPT2g/ placebo | 6 months | Randomized, double-blind, placebo-controlled clinical trial | 111 adults with NAFLD | no significant change was seen in the primary endpoint of hepatic fat fraction with respect to placebo | Blood | AST and gGTP improved | 14 |
|  | 3000 | 4 weeks | single-center, randomized, placebo-controlled, double-blind, phase I trial | 20 individuals with Parkinson's disease | NR therapy was well tolerated with no moderate or severe adverse events, and no significant difference in mild adverse events | Blood NAD+ levels | NR greatly augmented the blood NAD metabolome with up to 5-fold increase in blood NAD+ levels. | 15 |
|  | 150–500 | 2 years | A single-arm, open-label clinical trial | 14 patients with Ataxia Telangiectasia | The total scores in the neuromotor test panels improved, primarily driven by improvements in coordination subscores and eye movements | Blood NAD+ levels | NAD+ concentrations increased | 16 |
|  | max 1000 | 10 weeks | placebo-controlled randomized pilot study | 20 older adults with mild cognitive impairment | MoCA (Montreal Cognitive Assessment) and other neurocognitive and psychometric metrics remained stable throughout the study. | blood NAD+ levels; | NR significantly increased blood NAD+ concentrations | 17 |

NR, Nicotinamide Riboside; PBMNs, Peripheral Blood Mononuclear Cells; NAD, Nicotinamide Adenine Dinucleotide; NAM, Nicotinamide; MR, Magnetic Resonance; DXA, Dual-energy X-ray Absorptiometry; HECP, Hepatic Encephalopathy; cfPWV, Carotid-Femoral Pulse Wave Velocity; PWV, Pulse Wave Velocity; IVGTT, Intravenous Glucose Tolerance Test; HOMA, Homeostasis Model Assessment; OGTT, Oral Glucose Tolerance Test; 1H-MR, Proton Magnetic Resonance; PT, Pterostilbene; MuSC, Muscle Stem Cell; eGFR, Estimated Glomerular Filtration Rate; VO2, Volume of Oxygen; NRPT, Nicotinamide Riboside and Pterostilbene; NAFLD, Non-Alcoholic Fatty Liver Disease; AST, Aspartate Aminotransferase; gGTP, Gamma-Glutamyl Transpeptidase

**Supplementary Table 2. Clinical studies of NAD^+^ intermediate metabolites**

| Precursor | Dose | Periods | Study design | n | Results | Evaluation method | NAD⁺ metabolism | Supplementally References |
| --- | --- | --- | --- | --- | --- | --- | --- | --- |
| Nicotinic acid | Increase every 4 weeks from 250 to 1000 | Control group 4 months / mitochondrial myopathy group 10 months | non-randomized, open label | 8 healthy volunteers/ 5 patients with mitochondrial myopathy | Mitochondrial function and volume increased, improved muscle mass and strength improved, plasma adiponectin level increased, | Lateral vastus muscle biopsy, muscle strength test, MR spectroscopy, bioimpedance | PBMNs NAD⁺ increased | 18 |
|  |  |  |  |  | visceral and intrahepatic fat mass decreased only in mitochondrial myopathy group |  |  |  |
|  |  |  |  |  |  |  |  |  |
|  | 200 | Single | non-randomized, open label | 6 healthy volunteers | NAM pharmacokinetics, No side effects, TG decrease trend | Blood, Urine, LCMS/MS | NR and NAD⁺ in whole blood increased | 19 |
| Nicotinamide | 1000  3000 | Perioperative  3 days | Randomized, double-blind, placebo-controlled | 36 patients with cardiac surgery | Suppression of onset of postoperative acute kidney injury,  Markers of myocardial damage | Blood, urine | NAD⁺ in whole blood and NAM metabolite in urine increased | 20 |
|  | 500 –1750 | 12 weeks | 1 arm non-randomized | 65 patients on HD | P, intact PTH, LDL-C↓ HDL-C↑ | Blood | NAM metabolite in urine increased | 21 |
| NMN | 100/ 250/ 500 | Single administration | 1 arm non-randomized | 10 healthy volunteers | NMN Pharmacokinetics, No side effects | Blood, vital, ophthalmologic examination, sleep quality | PBMNs NAD⁺ increased | 22 |
|  | 1000/2000 | 14 days | double-blind, placebo-controlled study | 32 overweight or obese adults (55-80 yrs) | Blood NMN concentrations were significantly higher compared to placebo | Blood NAD levels | Blood levels of NAD metabolites elevated | 23 |
|  | 250 | 10 weeks | randomized, placebo-controlled, double-blind trial | 25 prediabetic women | Insulin-stimulated glucose disposal, assessed by using the hyperinsulinemic-euglycemic clamp, and skeletal muscle insulin signaling (phosphorylation of AKT and mTOR) | Plasma/PBMC | NAD+ content increased | 24 |
|  | 250 | 12 weeks | randomized, double-blind, placebo-controlled, parallel-group clinical trial | 36 healthy middle-aged participants | PWV values indicating arterial stiffness tended to decrease | Serum/PWV | NAM levels in the NMN intake group were significantly increased | 25 |
|  | 1000 | 28days | Randomized | 30 overweight or obese adults | Body weight, diastolic blood pressure, TC, LDL-C, and nHDL-C decreased significantly more in the MIB-626 group than placebo. | Blood, BP | Circulating concentrations of NAD and its metabolites increased | 26 |

NAD, Nicotinamide Adenine Dinucleotide; MR, Magnetic Resonance; PBMNs, Peripheral Blood Mononuclear Cells; NAM, Nicotinamide; TG, Triglycerides; LCMS/MS, Liquid Chromatography-Tandem Mass Spectrometry; PTH, Parathyroid Hormone; LDL-C, Low-Density Lipoprotein Cholesterol; HDL-C, High-Density Lipoprotein Cholesterol; PBMC, Peripheral Blood Mononuclear Cell; TC, Total Cholesterol; nHDL-C, non-HDL Cholesterol; BP, Blood Pressure

**Supplementary Table 3. Adverse events observed in the patients**

| **NR treatment period** | | | |  |  |  |  |
| --- | --- | --- | --- | --- | --- | --- | --- |
|  |  | **Mild** | |  |  |  |  |
| System organ class (SOC) | Preferred term (PT) | Number of patients | Number of events |  |  |  |  |
| General/systemic disorders and administration site conditions |  | 1 | 1 |  |  |  |  |
|  | Pain | 1 | 1 |  |  |  |  |
| Hepatobiliary system disorder |  | 1 | 1 |  |  |  |  |
|  | Abnormal liver function | 1 | 1 |  |  |  |  |
| Skin and subcutaneous tissue disorder |  | 1 | 4 |  |  |  |  |
|  | Subcutaneous hemorrhage | 1 | 1 |  |  |  |  |
|  | Hyperkeratosis | 1 | 1 |  |  |  |  |
|  | Pruritic rash | 1 | 1 |  |  |  |  |
|  | Skin ulcer | 1 | 1 |  |  |  |  |
| Vascular disorder |  | 1 | 1 |  |  |  |  |
|  | Hypotension | 1 | 1 |  |  |  |  |
|  |  |  |  |  |  |  |  |
| **Placebo treatment period** | | | | | | | |
|  |  | **Mild** | | **Moderate** | | **Severe** | |
| System organ class (SOC) | Preferred term (PT) | Number of patients | Number of events | Number of patients | Number of events | Number of patients | Number of events |
| Ocular disorders |  | 1 | 1 |  |  |  |  |
|  | Increased tear flow | 1 | 1 |  |  |  |  |
| Gastrointestinal disturbances |  | 1 | 1 |  |  |  |  |
|  | Diarrhea | 1 | 1 |  |  |  |  |
| Infectious and parasitic diseases |  | 1 | 1 |  |  | 1 | 1 |
|  | Pneumonia |  |  |  |  | 1 | 1 |
|  | COVID-19 | 1 | 1 |  |  |  |  |
| Injury, Poisoning and Treatment Complications |  | 1 | 1 |  |  |  |  |
|  | Chilblains | 1 | 1 |  |  |  |  |
| Musculoskeletal and Connective Tissue Disorders |  | 1 | 1 |  |  |  |  |
|  | Synovial fluid cysts | 1 | 1 |  |  |  |  |
| Renal and urinary tract disorders |  | 2 | 2 | 1 | 1 |  |  |
|  | Frequent urination | 1 | 1 |  |  |  |  |
|  | Renal enlargement | 1 | 1 |  |  |  |  |
|  | Renal masses |  |  | 1 | 1 |  |  |
| Skin and subcutaneous tissue disorders |  | 3 | 3 |  |  |  |  |
|  | Alopecia | 1 | 1 |  |  |  |  |
|  | Skin pain | 1 | 1 |  |  |  |  |
|  | Pruritus | 1 | 1 |  |  |  |  |

Abbreviations: NR, nicotinamide riboside

**Supplementary Table 4. Serum lipoprotein particle number per unit volume in each fraction at baseline measured using high-performance liquid chromatography**

|  |  | Fraction | n | Median  (nM) | Q1  (nM) | Q3  (nM) |
| --- | --- | --- | --- | --- | --- | --- |
| VLDL | Large VLDL | G3 | 6 | 2.4 | 1.0 | 5.0 |
|  |  | G4 | 6 | 10 | 4.9 | 15.8 |
|  |  | G5 | 6 | 38.1 | 19.9 | 41.1 |
|  | Medium VLDL | G6 | 6 | 48.6 | 29.1 | 59.3 |
|  | Small VLDL | G7 | 6 | 35.4 | 28.6 | 45.1 |
| LDL | Large LDL | G8 | 6 | 212.7 | 174.0 | 254.3 |
|  | Medium LDL | G9 | 6 | 598.7 | 489.3 | 693.8 |
|  | Small LDL | G10 | 6 | 235.2 | 209.0 | 283.1 |
|  | Very small LDL | G11 | 6 | 68 | 59.1 | 79.8 |
|  |  | G12 | 6 | 8.5 | 6.6 | 10.2 |
|  |  | G13 | 6 | 29.2 | 27.6 | 32.4 |
| HDL | Very large HDL | G14 | 6 | 33.5 | 18.1 | 40.9 |
|  |  | G15 | 6 | 179.2 | 149.3 | 214.2 |
|  | Large HDL | G16 | 6 | 2012.1 | 1603.3 | 2687.4 |
|  | Medium HDL | G17 | 6 | 5158.7 | 4993.9 | 5878.6 |
|  | Small HDL | G18 | 6 | 5268.6 | 4940.8 | 5616.3 |
|  | Very small HDL | G19 | 6 | 1750.9 | 1423.7 | 1768.3 |
|  |  | G20 | 6 | 961.3 | 748.5 | 1037.3 |

The lipoprotein particle is determined depend on particle size. The range of VLDL is 30–80 nm. The range of LDL is 16–30 nm. The range of HDL is 8–16 nm.

Abbreviations: nM, nanomolar; VLDL, very low-density lipoprotein; LDL, low-density lipoprotein; HDL, high-density lipoprotein; nm, nanometer

**Supplementary Table 5. High-performance liquid chromatography measurements of changes in serum lipoprotein particle number per unit volume across each fraction compared to baseline**

|  |  | Fraction | n | NR  (nM) | Placebo  (nM) | *P*-value |
| --- | --- | --- | --- | --- | --- | --- |
| VLDL | Large VLDL | G3 | 6 | -0.4 | 0.5 | 0.590 |
|  |  | G4 | 6 | -1.4 | 1.5 | 0.590 |
|  |  | G5 | 6 | -3.1 | 4.3 | 0.590 |
|  | Medium VLDL | G6 | 6 | 0.3 | -1.1 | 0.940 |
|  | Small VLDL | G7 | 6 | 7.5 | 13 | 0.400 |
| LDL | Large LDL | G8 | 6 | 42 | 29.6 | 0.940 |
|  | Medium LDL | G9 | 6 | -34.1 | 100.6 | 0.240 |
|  | Small LDL | G10 | 6 | -18.2 | 50.4 | 0.240 |
|  | Very small LDL | G11 | 6 | -1.7 | 11.6 | 0.310 |
|  |  | G12 | 6 | 0.6 | 4 | 1.000 |
|  |  | G13 | 6 | 3.3 | 3.7 | 0.820 |
| HDL | Very large HDL | G14 | 6 | 13 | 6.9 | 0.590 |
|  |  | G15 | 6 | 54.6 | 10.7 | 0.004 |
|  | Large HDL | G16 | 6 | 990.7 | -29.4 | 0.040 |
|  | Medium HDL | G17 | 6 | 581.5 | 228.1 | 0.820 |
|  | Small HDL | G18 | 6 | 137.4 | 500.1 | 0.390 |
|  | Very small HDL | G19 | 6 | 182.9 | 264.2 | 0.590 |
|  |  | G20 | 6 | 113.7 | 137 | 1.000 |

The lipoprotein particle is determined depending on particle size. The range of VLDL is 30–80 nm. The range of LDL is 16–30 nm. The range of HDL is 8–16 nm.

Abbreviations: nM, nanomolar; VLDL, very low-density lipoprotein; LDL, low-density lipoprotein; HDL, high-density lipoprotein, nm, nanometer

**Supplementary Table 6. Individual patient characteristics at baseline**

|  | Patient 1 | Patient 5 | Patient 6 | Patient 8 | Patient 9 | Patient 10 |
| --- | --- | --- | --- | --- | --- | --- |
| Allocation | NR preceding group | NR preceding group | Placebo preceding group | NR preceding group | NR preceding group | NR preceding group |
| Age (years) | 48 | 39 | 46 | 49 | 39 | 47 |
| Sex | Male | Female | Female | Female | Male | Male |
| Height (cm) | 161 | 146 | 152 | 148.7 | 166.9 | 169 |
| Body weight (kg) | 38.5 | 31.2 | 50 | 27 | 53 | 45 |
| BMI (kg/m²) | 14.9 | 14.6 | 21.6 | 12.2 | 19.0 | 15.8 |
| Waist circumference (cm) | 67 | 64 | 84 | 12.2 | 19.0 | 15.8 |
| Systolic blood pressure (mmHg) | 139 | 98 | 147 | 101 | 113 | 87 |
| Diastolic blood pressure (mmHg) | 71 | 48 | 84 | 62 | 69 | 55 |
| Pulse (bpm) | 74 | 65 | 73 | 78 | 86 | 72 |
| Right grip strength (kg) | 20.4 | 15 | 11.7 | 13.4 | 29.8 | 27.1 |
| Left grip strength (kg) | Not measurable | 15.4 | 10.4 | 11.3 | 27.7 | 22.1 |
| Walking speed (m/second) | 1.26 | 0.83 | 1.43 | 1.21 | 1.62 | Not measurable |
| Visceral fat area at umbilical height (cm^2^) | 20.6 | 58.9 | 107.3 | 19.7 | 125.5 | 205.8 |
| Skeletal muscle index (kg/m^2^) | 4.31 | 3.68 | 5.16 | 2.87 | 5.40 | 3.87 |
| Right heel pad thickness (mm) | 5.9 | 6.4 | 7.5 | 7.0 | 8.2 | 4.4 |
| Left heel pad thickness (mm) | 6.1 | 6.5 | 7.6 | 6.0 | 11.7 | 3.3 |
| Right CAVI | 9.5 | 6.8 | 7.8 | 8.5 | 7.2 | 8.6 |
| Left CAVI | 9.6 | 7.0 | 8.2 | 8.6 | 7.3 | 8.7 |
| Right ABI | 1.16 | 1.10 | 1.01 | 0.89 | 1.08 | 0.81 |
| Left ABI | 1.12 | 1.00 | 1.06 | 0.90 | 1.08 | 0.96 |
| Numerical Rating Scale | 3 | 3 | 1 | 5 | 3 | 7 |
| Diabetes mellitus | Yes | No | No | No | Yes | Yes |
| Dyslipidemia | Yes | No | Yes | Yes | Yes | Yes |
| Hypertension | No | Yes | No | No | No | No |
| Fatty liver | No | Yes | Yes | No | No | Yes |
| Cerebral hemorrhage | No | No | No | No | Yes | No |
| Cerebral infarction | No | No | No | No | No | No |
| Angina pectoris or myocardial infarction | No | Yes | No | No | No | No |
| Arterio-sclerosis obliterans | No | No | No | Yes | No | Yes |
| Malignancy | No | No | No | No | No | No |
| Hypothyroidism | Yes | Yes | Yes | Yes | No | Yes |
| Barthel Index | 95 | 100 | 100 | 100 | 100 | 85 |
| Gray hair or baldness | Yes | Yes | Yes | Yes | Yes | Yes |
| Bilateral cataract | Yes | Yes | Yes | Yes | Yes | Yes |
| Skin atrophy or hardening | Yes | Yes | Yes | Yes | Yes | Yes |
| Skin ulcer | Yes | No | No | Yes | No | Yes |
| Soft tissue calcification | Yes | Yes | Yes | Yes | Yes | Yes |
| Bird-like face | Yes | Yes | No | Yes | Yes | Yes |
| High-pitched voice | Yes | Yes | No | Yes | Yes | Yes |
| Limb amputation | No | No | No | No | No | No |
| Family history of Werner syndrome | No | No | No | No | No | No |
| Consanguineous marriage | Yes | No | No | No | No | No |
| Genetic testing | Yes | Yes | Yes | Yes | Yes | Yes |
| ***WRN* mutation type** | mutation 4/4 | mutation 4/23 | mutation 4/4 | mutation 20/21 | mutation 4/4 | mutation 4/19 |
| **Blood and urine test** |  |  |  |  |  |  |
| WBC (/μL) | 4600 | 7200 | 5100 | 6200 | 6300 | 5500 |
| RBC (x10^4^/μL) | 446 | 376 | 472 | 445 | 442 | 379 |
| Hemoglobin (g/dL) | 12.8 | 11.2 | 15 | 13.4 | 14.1 | 12.5 |
| Hematocrit (%) | 39.4 | 33.4 | 44.3 | 40 | 42.1 | 37.6 |
| Platelet (x10^4^/μL) | 20.6 | 30.8 | 23.1 | 24.4 | 12.8 | 15.9 |
| AST (U/L) | 71 | 32 | 30 | 26 | 25 | 27 |
| ALT (U/L) | 63 | 43 | 39 | 21 | 25 | 37 |
| LDH (U/L) | 281 | 188 | 219 | 188 | 171 | 123 |
| ALP (U/L) | 91 | 62 | 106 | 78 | 108 | 78 |
| γGTP (U/L) | 75 | 112 | 97 | 40 | 52 | 31 |
| CPK (U/L) | 353 | 91 | 122 | 91 | 60 | 70 |
| T-bilirubin (mg/dL) | 0.7 | 0.3 | 0.6 | 0.7 | 0.9 | 0.5 |
| C-bilirubin (mg/dL) | 0.1 | 0 | 0.1 | 0.1 | 0.1 | 0.1 |
| Total protein (g/dL) | 8.3 | 7.4 | 7.3 | 7.5 | 7.7 | 6.9 |
| Serum albumin (g/dL) | 4.3 | 4.6 | 4.5 | 4.5 | 4.6 | 4.2 |
| Uric acid (mg/dL) | 4 | 7.5 | 4.8 | 5.8 | 2.9 | 5.2 |
| Na (mmol/L) | 142 | 141 | 140 | 142 | 139 | 144 |
| K (mmol/L) | 4.1 | 3.9 | 3.9 | 4 | 3.9 | 4.3 |
| Cl (mmol/L) | 105 | 105 | 105 | 106 | 106 | 109 |
| Total cholesterol (mg/dL) | 209 | 200 | 236 | 194 | 186 | 119 |
| Triglyceride (mg/dL) | 69 | 71 | 109 | 148 | 142 | 235 |
| LDL-cholesterol (mg/dL) | 121 | 114 | 154 | 97 | 95 | 47 |
| HDL-cholesterol (mg/dL) | 79 | 79 | 66 | 79 | 59 | 48 |
| Serum glucose (mg/dL) | 105 | 116 | 98 | 91 | 120 | 123 |
| HbA1c (%) | 8.5 | 5.6 | 5.2 | 5.6 | 5.7 | 5.2 |
| CRP (mg/dL) | 0.14 | 0.06 | 0.15 | 0.02 | 0.07 | 0.54 |
| NAD^+^ (unit) | 0.06 | 0.04 | 0.04 | 0.07 | 0 | 0 |
| BUN (mg/dL) | 26 | 20 | 10 | 14 | 16 | 10 |
| Serum creatinine (mg/dL) | 0.56 | 0.48 | 0.53 | 0.75 | 0.59 | 0.82 |
| eGFR (mL/min/1.73 m^2^) | 120.4 | 111.8 | 96.3 | 64.3 | 120.7 | 79.8 |
| Urine albumin (mg/gCr) | 11.2 | 10 | 5 | 8.5 | 4.4 | 13.9 |
| Urine NAG (U/L) | 11.1 | 2.5 | 3.8 | 2.5 | 16.4 | 1.6 |
| β2-microglobulin (mg/L) | 227 | 39 | 73 | 47 | 39 | 220 |
| L-FABP (μg/gCr) | 4.3 | 3.58 | 2.52 | 2.81 | 3.22 | 3.51 |
| Kim-1 (ng/mL) | 0.603 | 0.388 | 0.867 | 0.171 | 2.37 | 0.162 |

Abbreviations: FAS, full analysis set; PPS, per-protocol set; SAF, safety analysis set; NR, nicotinamide riboside; SD, standard deviation; IQR, interquartile range; mutation 4, c.3139-1G>C; mutation 6, c.1105C>T; mutation 19, c.2344_2351delGGTGAACT; mutation 20, c.2630G>C; mutation 21, c.2630G>T; mutation 23, c.3383+1G>T; CAVI, cardio-ankle vascular index; ABI, ankle-brachial index; WBC, white blood cell; RBC, red blood cell; AST, aspartate aminotransferase; ALT, alanine aminotransferase; LDH, lactate dehydrogenase; ALP, alkaline phosphatase; γGTP, gamma-glutamyl transferase; CPK, creatine phosphokinase; T-bilirubin, total bilirubin; C-bilirubin, conjugated bilirubin; LDL, low-density lipoprotein; HDL, high-density lipoprotein; HbA1c, glycated hemoglobin; CRP, C-reactive protein; NAD, nicotinamide adenine dinucleotide; BUN, blood urea nitrogen; eGFR, estimated glomerular filtration rate; NAG, N-acetyl-β-D-glucosaminidase; L-FABP, liver-type fatty acid-binding protein; Kim-1, kidney injury molecule-1; NA: not available

**Supplementary Table 7. Changes in clinical parameters after NR phase from baseline**

| **Biometric measurements** | | | |  |  |  |
| --- | --- | --- | --- | --- | --- | --- |
|  | Patient 1 | Patient 5 | Patient 6 | Patient 8 | Patient 9 | Patient 10 |
| Systolic blood pressure (mmHg) | -3 | 43 | -13 | -9 | -12 | 13 |
| Diastolic blood pressure (mmHg) | 8 | 23 | 11 | 2 | -8 | 6 |
| Right grip strength (kg) | 1.4 | 2 | -0.6 | 1.1 | -2.5 | -3.4 |
| Left grip strength (kg) | Not measurable | 0.4 | 1.8 | 0.9 | -2.7 | -2.3 |
| Walking speed (m/second) | -0.1 | 0.35 | 0.02 | -0.28 | 0.06 | Not measurable |
| Visceral fat area at umbilical height (cm^2^) | 7.05 | 19.74 | -8.88 | 8.8 | -44.45 | -104.71 |
| Skeletal muscle index (kg/m^2^) | 0.14 | -0.01 | -0.22 | 0.01 | 0.21 | -0.01 |
| Right heel pad thickness (mm) | 0 | 1.9 | -1.4 | -0.9 | 0.4 | -1.4 |
| Left heel pad thickness (mm) | 1.4 | 1.2 | -0.6 | 0.5 | -2.7 | 1.1 |
| Right CAVI | -0.3 | -0.2 | 1.1 | -1 | 1.2 | -1.2 |
| Left CAVI | -0.3 | -0.5 | 0.6 | -1.1 | 1.1 | -1.3 |
| Right ABI | -0.12 | -0.01 | 0.09 | -0.2 | 0 | -0.2 |
| Left ABI | -0.07 | 0.06 | 0.01 | -0.19 | 0 | -0.27 |
| Numerical Rating Scale | -4 | 2 | 0 | 7 | -3 | -2 |
| **Blood and urine test** | | | |  |  |  |
| WBC (/μL) | -400 | -300 | 900 | 1100 | -1100 | 1300 |
| RBC (x10^4^/μL) | 36 | -24 | 5 | 3 | -4 | 24 |
| Hemoglobin (g/dL) | 0.6 | -0.4 | 0.6 | 0.1 | 0.4 | 0.6 |
| Hematocrit (%) | 2.1 | -1.5 | 2.1 | 0.1 | 1.4 | 2.1 |
| Platelet (x10^4^/μL) | -2.4 | -1.8 | 1.1 | -0.8 | 1.5 | 1.2 |
| AST (U/L) | -24 | 38 | 22 | 31 | -3 | 1 |
| ALT (U/L) | -24 | 51 | 78 | 34 | -4 | -9 |
| LDH (U/L) | 20 | 41 | 23 | 28 | -13 | 0 |
| ALP (U/L) | 12 | 45 | 59 | 0 | -9 | 0 |
| γGTP (U/L) | -46 | 272 | 238 | 17 | -20 | -11 |
| CPK (U/L) | 171 | 84 | 20 | 39 | 16 | 9 |
| T-bilirubin (mg/dL) | 0.1 | 0 | 0 | 0 | -0.1 | 0 |
| C-bilirubin (mg/dL) | 0 | 0.1 | 0 | 0 | 0 | 0 |
| Total protein (g/dL) | -0.2 | 0 | 0.9 | 0.3 | -0.1 | -0.1 |
| Serum albumin (g/dL) | 0 | 0 | 0.6 | 0.3 | -0.1 | -0.1 |
| Uric acid (mg/dL) | 1.3 | -0.8 | 0.4 | 0.1 | 0.2 | -0.1 |
| Na (mmol/L) | -2 | -3 | -1 | -1 | -1 | -3 |
| K (mmol/L) | -0.1 | 0.4 | 0 | 0.3 | 0.1 | -0.2 |
| Cl (mmol/L) | -1 | -3 | -2 | -3 | 0 | -1 |
| Total cholesterol (mg/dL) | -17 | 36 | 46 | 23 | 46 | -8 |
| Triglyceride (mg/dL) | -16 | 53 | -14 | -47 | 17 | 48 |
| LDL-cholesterol (mg/dL) | -16 | 15 | 19 | 13 | 33 | 4 |
| HDL-cholesterol (mg/dL) | -4 | 19 | 29 | 15 | 17 | -11 |
| Serum glucose (mg/dL) | -5 | -6 | 5 | 14 | 12 | -31 |
| HbA1c (%) | -2.2 | 0.5 | 0.1 | 0.1 | -0.2 | -0.1 |
| CRP (mg/dL) | -0.03 | 0.04 | 0.07 | 0.01 | -0.03 | -0.43 |
| NAD^+^ (unit) | -0.04 | 0.1 | 0.09 | 0.13 | 0.04 | 0.04 |
| BUN (mg/dL) | -7 | -4 | -3 | 2 | 1 | 3 |
| Serum creatinine (mg/dL) | 0.05 | -0.03 | -0.01 | 0.06 | -0.02 | -0.06 |
| eGFR (mL/min/1.73 m^2^) | -11.4 | 7.2 | 1.4 | -5.2 | 3.8 | 7 |
| Urine albumin (mg/gCr) | -7.2 | -0.8 | 8.5 | 2.3 | 1.8 | 66.5 |
| Urine NAG (U/L) | -6.9 | -1.6 | -3.1 | -1 | 4.7 | 1.6 |
| β2-microglobulin (mg/L) | -148 | -10 | -44 | -15 | 40 | 339 |
| L-FABP (μg/gCr) | Not measurable | -0.57 | 0.96 | 2.53 | Not measurable | 1.52 |
| Kim-1 (ng/mL) | -0.421 | -0.233 | -0.712 | -0.016 | -0.73 | 1.308 |

Abbreviations: NR, nicotinamide riboside; SD, standard deviation; IQR, interquartile range; CAVI, cardio-ankle vascular index; ABI, ankle-brachial index; WBC, white blood cell; RBC, red blood cell; AST, aspartate aminotransferase; ALT, alanine aminotransferase; LDH, lactate dehydrogenase; ALP, alkaline phosphatase; γGTP, gamma-glutamyl transferase; CPK, creatine phosphokinase; T-bilirubin, total bilirubin; C-bilirubin, conjugated bilirubin; LDL, low-density lipoprotein; HDL, high-density lipoprotein; HbA1c, glycated hemoglobin; CRP, C-reactive protein; NAD, nicotinamide adenine dinucleotide; BUN, blood urea nitrogen; eGFR, estimated glomerular filtration rate; NAG, N-acetyl-β-D-glucosaminidase; L-FABP, liver-type fatty acid-binding protein; Kim-1, kidney injury molecule-1; NA: not available

**Supplementary Table 8. Changes in clinical parameters after placebo phase from baseline**

| **Biometric measurements** | | | |  |  |  |
| --- | --- | --- | --- | --- | --- | --- |
|  | Patient 1 | Patient 5 | Patient 6 | Patient 8 | Patient 9 | Patient 10 |
| Systolic blood pressure (mmHg) | -3 | 28 | -5 | -2 | 2 | -2 |
| Diastolic blood pressure (mmHg) | 2 | 25 | -13 | 2 | -4 | 0 |
| Right grip strength (kg) | -0.8 | -0.2 | -2 | -0.1 | 1 | -6.6 |
| Left grip strength (kg) | Not measurable | -3.2 | -2.4 | 0.9 | 0.5 | -1.1 |
| Walking speed (m/second) | -0.37 | 0.28 | -0.01 | -0.1 | -0.03 | Not measurable |
| Visceral fat area at umbilical height (cm^2^) | 20.43 | 14.62 | 3.69 | 22.58 | 41.55 | -93.54 |
| Skeletal muscle index (kg/m^2^) | 0.19 | -0.21 | -0.06 | -0.06 | 0.52 | -0.15 |
| Right heel pad thickness (mm) | -0.1 | 0.8 | -1 | -1 | -1.7 | -0.6 |
| Left heel pad thickness (mm) | 0.4 | 1.1 | -0.8 | -0.4 | -4.9 | 0.1 |
| Right CAVI | 0.3 | 2.2 | -0.6 | -0.1 | 0.7 | 0.9 |
| Left CAVI | 0.1 | 1.9 | -0.8 | -0.2 | 0.7 | 0.8 |
| Right ABI | -0.12 | 0.18 | -0.07 | -0.01 | 0.06 | 0.1 |
| Left ABI | -0.16 | 0.1 | -0.09 | -0.05 | 0.08 | 0.1 |
| Numerical Rating Scale | 1 | 1 | -5 | 0 | -2 | -1 |
| Blood and urine test | | | |  |  |  |
| WBC (/μL) | 100 | -700 | 800 | 400 | -100 | 2800 |
| RBC (x10^4^/μL) | 68 | 7 | -4 | -3 | -18 | -27 |
| Hemoglobin (g/dL) | 1.6 | 0.1 | 0.3 | -0.3 | -0.4 | -1.3 |
| Hematocrit (%) | 5.7 | 1.5 | 0.6 | 0.1 | -1.4 | -2.3 |
| Platelet (x10^4^/μL) | -2.2 | 4.3 | -0.4 | 0 | 1.2 | -1.1 |
| AST (U/L) | -41 | 0 | 14 | 8 | 0 | -3 |
| ALT (U/L) | -37 | 0 | 49 | 16 | 2 | -8 |
| LDH (U/L) | -97 | 7 | 1 | 1 | 4 | 37 |
| ALP (U/L) | 7 | 20 | 34 | 8 | 11 | 7 |
| γGTP (U/L) | -41 | 60 | 105 | 8 | -9 | 2 |
| CPK (U/L) | -229 | 3 | 38 | 5 | 29 | -13 |
| T-bilirubin (mg/dL) | 0 | 0 | 0.2 | -0.2 | -0.2 | -0.1 |
| C-bilirubin (mg/dL) | 0 | 0.1 | 0 | -0.1 | 0 | 0 |
| Total protein (g/dL) | 0.2 | 0.4 | 0.4 | 0.2 | 0 | 0.3 |
| Serum albumin (g/dL) | 0.4 | 0.4 | 0.3 | 0.3 | -0.1 | -0.3 |
| Uric acid (mg/dL) | 2.2 | 0 | 0.5 | -0.1 | 0.5 | 1.7 |
| Na (mmol/L) | -1 | -1 | 1 | -2 | 0 | -2 |
| K (mmol/L) | 0.8 | 0.5 | 0.2 | 0.3 | 0 | 0.3 |
| Cl (mmol/L) | -1 | -3 | 0 | -2 | 0 | 1 |
| Total cholesterol (mg/dL) | 29 | 33 | 20 | 19 | 33 | -20 |
| Triglyceride (mg/dL) | 52 | -5 | 4 | 63 | -9 | -100 |
| LDL-cholesterol (mg/dL) | 32 | 16 | 4 | 14 | 39 | -15 |
| HDL-cholesterol (mg/dL) | -8 | 12 | 14 | -8 | 6 | 3 |
| Serum glucose (mg/dL) | 38 | -2 | -1 | 5 | 5 | 5 |
| HbA1c (%) | -2 | 0.3 | -0.1 | 0 | 0.1 | 0.4 |
| CRP (mg/dL) | -0.1 | -0.01 | 0.06 | -0.01 | 0.02 | -0.21 |
| NAD^+^ (unit) | 0 | 0.03 | 0.09 | -0.02 | 0 | 0 |
| BUN (mg/dL) | -8 | -5 | 1 | 2 | -3 | 11 |
| Serum creatinine (mg/dL) | 0.11 | 0.02 | 0.02 | 0 | 0.02 | 0.38 |
| eGFR (mL/min/1.73 m^2^) | -22 | -5.6 | -3.8 | -0.4 | -5.1 | -27.5 |
| Urine albumin (mg/gCr) | -6.4 | 5 | 3.3 | 6.2 | 0.9 | 79 |
| Urine NAG (U/L) | -8.4 | -1.8 | -1.5 | -0.9 | -10.4 | 4.5 |
| β2-microglobulin (mg/L) | -190 | -10 | -37 | 1 | 25 | 2621 |
| L-FABP (μg/gCr) | -2.11 | -0.3 | 0.68 | 1.22 | -1.06 | 10.69 |
| Kim-1 (ng/mL) | -0.125 | -0.233 | -0.295 | -0.016 | -1.24 | 14.338 |

Abbreviations: NR, nicotinamide riboside; SD, standard deviation; IQR, interquartile range; CAVI, cardio-ankle vascular index; ABI, ankle-brachial index; WBC, white blood cell; RBC, red blood cell; AST, aspartate aminotransferase; ALT, alanine aminotransferase; LDH, lactate dehydrogenase; ALP, alkaline phosphatase; γGTP, gamma-glutamyl transferase; CPK, creatine phosphokinase; T-bilirubin, total bilirubin; C-bilirubin, conjugated bilirubin; LDL, low-density lipoprotein; HDL, high-density lipoprotein; HbA1c, glycated hemoglobin; CRP, C-reactive protein; NAD, nicotinamide adenine dinucleotide; BUN, blood urea nitrogen; eGFR, estimated glomerular filtration rate; NAG, N-acetyl-β-D-glucosaminidase; L-FABP, liver-type fatty acid-binding protein; Kim-1, kidney injury molecule-1; NA: not available

**Supplementary Table 9. Serum lipoprotein particle number per unit volume in each fraction measured using high-performance liquid chromatography at baseline**

|  |  | Fraction | Patient 1 | Patient 5 | Patient 6 | Patient 8 | Patient 9 | Patient 10 |
| --- | --- | --- | --- | --- | --- | --- | --- | --- |
| VLDL | Large VLDL | G3 | 0.56 | 2.56 | 4.98 | 0.98 | 2.30 | 7.11 |
|  |  | G4 | 1.66 | 10.64 | 15.84 | 4.88 | 9.42 | 23.86 |
|  |  | G5 | 14.54 | 41.06 | 37.60 | 19.85 | 38.53 | 55.28 |
|  | Medium VLDL | G6 | 26.10 | 52.85 | 44.27 | 29.14 | 82.45 | 59.30 |
|  | Small VLDL | G7 | 39.93 | 45.10 | 30.86 | 20.62 | 62.07 | 28.56 |
| LDL | Large LDL | G8 | 254.28 | 296.10 | 173.97 | 206.16 | 219.33 | 91.07 |
|  | Medium LDL | G9 | 627.13 | 837.61 | 570.18 | 693.82 | 489.32 | 327.93 |
|  | Small LDL | G10 | 208.95 | 283.07 | 254.52 | 285.28 | 173.46 | 215.83 |
|  | Very small LDL | G11 | 65.18 | 83.31 | 70.74 | 79.81 | 53.29 | 59.05 |
|  |  | G12 | 6.58 | 5.97 | 8.52 | 10.22 | 8.38 | 13.05 |
|  |  | G13 | 29.65 | 32.37 | 28.84 | 32.61 | 27.61 | 24.42 |
| HDL | Very large HDL | G14 | 39.05 | 40.91 | 28.00 | 48.63 | 18.10 | 17.26 |
|  |  | G15 | 214.17 | 149.32 | 220.38 | 181.50 | 176.84 | 113.13 |
|  | Large HDL | G16 | 2687.40 | 1603.29 | 2709.56 | 2092.26 | 1931.87 | 672.51 |
|  | Medium HDL | G17 | 5078.89 | 5238.43 | 6097.44 | 5878.63 | 4993.90 | 3638.82 |
|  | Small HDL | G18 | 4668.26 | 5358.58 | 5616.33 | 5178.53 | 4940.77 | 5850.98 |
|  | Very small HDL | G19 | 1768.34 | 1760.54 | 1741.30 | 1423.73 | 1206.71 | 1807.60 |
|  |  | G20 | 921.32 | 1037.29 | 1001.25 | 1124.72 | 748.53 | 744.27 |

The lipoprotein particle is determined depending on particle size. The range of VLDL is 30–80 nm. The range of LDL is 16–30 nm. The range of HDL is 8–16 nm.

Abbreviations: nM, nanomolar; VLDL, very low-density lipoprotein; LDL, low-density lipoprotein; HDL, high-density lipoprotein; nm, nanometer

**Supplementary Table 10. Changes of serum lipoprotein particle number per unit volume in each fraction compared to baseline measured using high-performance liquid chromatography after NR phase**

|  |  | Fraction | Patient 1 | Patient 5 | Patient 6 | Patient 8 | Patient 9 | Patient 10 |
| --- | --- | --- | --- | --- | --- | --- | --- | --- |
| VLDL | Large VLDL | G3 | -0.37 | 3.24 | -0.34 | -3.23 | -0.41 | 1.06 |
|  |  | G4 | -0.77 | 9.79 | -3.10 | -8.83 | -2.05 | 3.97 |
|  |  | G5 | -1.65 | 19.32 | -10.17 | -10.42 | -4.46 | 11.43 |
|  | Medium VLDL | G6 | -0.42 | 9.02 | -21.57 | 1.25 | -4.05 | 0.94 |
|  | Small VLDL | G7 | -7.94 | 7.14 | 14.87 | 7.78 | -3.25 | 10.59 |
| LDL | Large LDL | G8 | -40.33 | 32.69 | 51.33 | 59.56 | -11.21 | 57.18 |
|  | Medium LDL | G9 | -50.78 | -17.51 | -78.01 | 78.48 | -65.55 | 99.02 |
|  | Small LDL | G10 | 8.68 | -26.02 | -68.23 | -10.37 | -41.19 | 23.45 |
|  | Very small LDL | G11 | -2.44 | -1.03 | -11.19 | 0.84 | -5.38 | 8.81 |
|  |  | G12 | 10.09 | 4.53 | 1.16 | -1.44 | 0.00 | -0.50 |
|  |  | G13 | 12.28 | 4.45 | 2.01 | 2.11 | -0.71 | 7.11 |
| HDL | Very large HDL | G14 | 35.39 | 20.48 | 13.38 | 12.62 | 8.18 | -2.67 |
|  |  | G15 | 56.06 | 51.90 | 65.88 | 53.08 | 31.59 | 145.60 |
|  | Large HDL | G16 | -175.53 | 1256.81 | 1152.43 | 828.99 | 804.32 | 1235.14 |
|  | Medium HDL | G17 | -286.43 | 850.48 | 900.25 | 545.75 | 38.71 | 617.31 |
|  | Small HDL | G18 | 381.01 | 862.51 | -36.37 | -79.35 | -588.70 | 311.08 |
|  | Very small HDL | G19 | 0.33 | 789.12 | 224.33 | 141.37 | 107.59 | 351.71 |
|  |  | G20 | 48.88 | 111.66 | 115.75 | 218.19 | -123.47 | 260.35 |

The lipoprotein particle is determined depending on particle size. The range of VLDL is 30–80 nm. The range of LDL is 16–30 nm. The range of HDL is 8–16 nm.

Abbreviations: nM, nanomolar; VLDL, very low-density lipoprotein; LDL, low-density lipoprotein; HDL, high-density lipoprotein; nm, nanometer

**Supplementary Table 11. Changes of serum lipoprotein particle number per unit volume in each fraction compared to baseline measured using high-performance liquid chromatography after placebo phase**

|  |  | Fraction | Patient 1 | Patient 5 | Patient 6 | Patient 8 | Patient 9 | Patient 10 |
| --- | --- | --- | --- | --- | --- | --- | --- | --- |
| VLDL | Large VLDL | G3 | 1.53 | 0.35 | -0.02 | 2.11 | -5.13 | 0.22 |
|  |  | G4 | 8.15 | 0.02 | -0.34 | 4.57 | -13.31 | -0.45 |
|  |  | G5 | 29.31 | 2.60 | -0.48 | 9.57 | -17.84 | 0.76 |
|  | Medium VLDL | G6 | 39.14 | -3.64 | -2.01 | 24.08 | -1.52 | -13.48 |
|  | Small VLDL | G7 | 13.68 | 0.70 | 17.58 | 22.24 | 5.32 | 12.39 |
| LDL | Large LDL | G8 | 14.47 | 44.37 | 57.14 | 44.69 | 2.14 | 53.31 |
|  | Medium LDL | G9 | 143.46 | 96.30 | -49.78 | 65.22 | -74.78 | 135.99 |
|  | Small LDL | G10 | 108.20 | 36.28 | -56.36 | 39.44 | -61.92 | 61.37 |
|  | Very small LDL | G11 | 20.61 | 14.04 | -7.03 | 10.93 | -15.51 | 12.33 |
|  |  | G12 | 7.93 | 0.80 | -1.51 | 4.71 | -5.49 | 3.47 |
|  |  | G13 | 11.03 | 2.29 | 2.12 | 5.46 | -7.11 | -1.01 |
| HDL | Very large HDL | G14 | 11.03 | 14.08 | 2.24 | 2.85 | 0.77 | 13.95 |
|  |  | G15 | 13.49 | 21.32 | 46.42 | 10.16 | 11.24 | -58.10 |
|  | Large HDL | G16 | -1178.75 | 742.00 | 875.24 | -413.22 | 629.50 | -205.70 |
|  | Medium HDL | G17 | 75.15 | 767.20 | 282.17 | -287.07 | 173.98 | 691.74 |
|  | Small HDL | G18 | 2043.92 | 1426.11 | 220.82 | 779.47 | -475.08 | 195.38 |
|  | Very small HDL | G19 | 237.33 | 969.53 | 258.92 | 269.45 | 13.44 | 325.67 |
|  |  | G20 | 213.22 | 244.02 | 86.74 | 29.00 | -8.93 | 187.20 |

The lipoprotein particle is determined depending on particle size. The range of VLDL is 30–80 nm. The range of LDL is 16–30 nm. The range of HDL is 8–16 nm.

Abbreviations: nM, nanomolar; VLDL, very low-density lipoprotein; LDL, low-density lipoprotein; HDL, high-density lipoprotein, nm, nanometer

**Supplementary Table 12. Inclusion and exclusion criteria**

| **Inclusion Criteria** | Target patients must meet all the following conditions:  1) Patients who have been diagnosed with Werner syndrome  2) Patients who have been explained the contents of the study using the explanatory document and given their written consent to participate in the study  3) Men or women aged > 20 years  4) Patients whose hematocrit value was clinically acceptable by the investigators  5) Patients deemed likely to comply with protocol requirements by the investigators |
| --- | --- |
| **Exclusion Criteria** | Those who meet any of the following conditions:  1) Serious illnesses determined by the investigators to have the potential to affect the interpretation of the data or safety or to make the protocol evaluation insecure (including patients with abnormal laboratory values)  2) Patients deemed to have significant dehydration by the investigators  3) Patients with pericardial effusion, ascites, and pleural effusion  4) Patient who participated in another clinical trial within 30 days before obtaining consent  5) Patients whose clinical signs at screening indicated the final stages of progression to Werner syndrome and are difficult to safely complete the study and its evaluated, including:  • Patients who received continuous or intermittent home oxygen therapy for 6 months before obtaining consent  • Patients who were hospitalized two or more times for pneumonia in the 12 months before obtaining consent  • Patients who had at least a 10% weight loss on medical record and did not recover. This included significant weight loss over the last 6 months (beyond estimated measurement error)  • CKD stage 5 and/or patients requiring peritoneal or hemodialysis  • Patients with liver function test values more than three times the upper normal limit  6) Patients with a history of hypersensitivity to nicotinamide riboside  7) Patients with a history of serious drug hypersensitivity or allergic reactions such as anaphylaxis  8) Patients with an estimated survival time of < 2 years at the end of life  9) Patients who could not comply with research requirements and procedures  10) Patients with malignant tumors or a history of malignancy  11) Patient whose tumor was suspected using chest X-ray examination  12) Patients with a positive fecal occult blood test (if positive, patients without neoplastic lesions by upper and lower gastrointestinal endoscopy could participate)  13) Pregnant women, nursing women, and patients who may be pregnant  14) Patients deemed inappropriate by the investigators  15) Patients taking nicotinamide riboside within 30 days of first screening visit |

Abbreviations: CKD, chronic kidney disease

**Supplementary References**

1. Trammell, S. A. J. et al. Nicotinamide riboside is uniquely and orally bioavailable in mice and humans. *Nat. Commun.* **7**, 12948 (2016). [10.1038/ncomms12948](https://doi.org/10.1038/ncomms12948), Pubmed:[27721479](https://www.ncbi.nlm.nih.gov/pubmed/27721479).

2. Airhart, S. E. et al. An open-label, non-randomized study of the pharmacokinetics of the nutritional supplement nicotinamide riboside (NR) and its effects on blood NAD^+^levels in healthy volunteers. *PLOS ONE* **12**, e0186459 (2017). [10.1371/journal.pone.0186459](https://doi.org/10.1371/journal.pone.0186459), Pubmed:[29211728](https://www.ncbi.nlm.nih.gov/pubmed/29211728).

3. Conze, D., Brenner, C. & Kruger, C. L. Safety and metabolism of long-term administration of NIAGEN (nicotinamide riboside chloride) in a randomized, double-blind, placebo-controlled clinical trial of healthy overweight adults. *Sci. Rep.* **9**, 9772 (2019). [10.1038/s41598-019-46120-z](https://doi.org/10.1038/s41598-019-46120-z), Pubmed:[31278280](https://www.ncbi.nlm.nih.gov/pubmed/31278280).

4. Dolopikou, C. F. et al. Acute nicotinamide riboside supplementation improves redox homeostasis and exercise performance in old individuals: a double-blind cross-over study. *Eur. J. Nutr.* **59**, 505−515 (2020). [10.1007/s00394-019-01919-4](https://doi.org/10.1007/s00394-019-01919-4), Pubmed:[30725213](https://www.ncbi.nlm.nih.gov/pubmed/30725213).

5. Dollerup, O. L. et al. A randomized placebo-controlled clinical trial of nicotinamide riboside in obese men: safety, insulin-sensitivity, and lipid-mobilizing effects. *Am. J. Clin. Nutr.* **108**, 343−353 (2018). [10.1093/ajcn/nqy132](https://doi.org/10.1093/ajcn/nqy132), Pubmed:[29992272](https://www.ncbi.nlm.nih.gov/pubmed/29992272).

6. Martens, C. R. et al. Chronic nicotinamide riboside supplementation is well-tolerated and elevates NAD+ in healthy middle-aged and older adults. *Nat. Commun.* **9**, 1286 (2018). [10.1038/s41467-018-03421-7](https://doi.org/10.1038/s41467-018-03421-7), Pubmed:[29599478](https://www.ncbi.nlm.nih.gov/pubmed/29599478).

7. Dollerup, O. L. et al. Effects of nicotinamide riboside on endocrine pancreatic function and incretin hormones in nondiabetic men with obesity. *J. Clin. Endocrinol. Metab.* **104**, 5703−5714 (2019). [10.1210/jc.2019-01081](https://doi.org/10.1210/jc.2019-01081), Pubmed:[31390002](https://www.ncbi.nlm.nih.gov/pubmed/31390002).

8. Dollerup, O. L. et al. Nicotinamide riboside does not alter mitochondrial respiration, content or morphology in skeletal muscle from obese and insulin-resistant men. *J. Physiol.* **598**, 731−754 (2020). [10.1113/JP278752](https://doi.org/10.1113/JP278752), Pubmed:[31710095](https://www.ncbi.nlm.nih.gov/pubmed/31710095).

9. Remie, C. M. E. et al. Nicotinamide riboside supplementation alters body composition and skeletal muscle acetylcarnitine concentrations in healthy obese humans. *Am. J. Clin. Nutr.* 112, 413–426 (2020). [10.1093/ajcn/nqaa072](https://doi.org/10.1093/ajcn/nqaa072), Pubmed:[32320006](https://www.ncbi.nlm.nih.gov/pubmed/32320006).

10. Elhassan, Y. S. et al. Nicotinamide riboside augments the aged human skeletal muscle NAD+ metabolome and induces transcriptomic and anti-inflammatory signatures. *Cell Rep.* **28**, 1717−1728.e6 (2019). 10.1016/j.celrep.2019.07.043, Pubmed:31412242.

11. Brakedal, B. et al. The NADPARK study: A randomized phase I trial of nicotinamide riboside supplementation in Parkinson's disease. *Cell Metab.* **34**, 396–407.e6 (2022). [10.1016/j.cmet.2022.02.001](https://doi.org/10.1016/j.cmet.2022.02.001), PMID: [35235774](http://www.ncbi.nlm.nih.gov/pubmed/35235774).

12. Jensen, J. B. et al. A randomized placebo-controlled trial of nicotinamide riboside and pterostilbene supplementation in experimental muscle injury in elderly individuals. *JCI Insight* **7**, e158314 (2022). [10.1172/jci.insight.158314](https://doi.org/10.1172/jci.insight.158314), PMID: [35998039](http://www.ncbi.nlm.nih.gov/pubmed/35998039), PMCID: [PMC9675477](https://www.ncbi.nlm.nih.gov/pmc/articles/PMC9675477).

13. Ahmadi, A. et al. Randomized crossover clinical trial of coenzyme Q10 and nicotinamide riboside in chronic kidney disease. *JCI Insight* **8**, e167274 (2023). [10.1172/jci.insight.167274](https://doi.org/10.1172/jci.insight.167274), PMID: [37159264](http://www.ncbi.nlm.nih.gov/pubmed/37159264), PMCID: [PMC10393227](https://www.ncbi.nlm.nih.gov/pmc/articles/PMC10393227).

14. Dellinger, R. W. et al. Nicotinamide riboside and pterostilbene reduces markers of hepatic inflammation in NAFLD: A double-blind, placebo-controlled clinical trial. *Hepatology* **78**, 863−877 (2023). [10.1002/hep.32778](https://doi.org/10.1002/hep.32778), PMID: [36082508](http://www.ncbi.nlm.nih.gov/pubmed/36082508).

15. Berven, H. et al. NR-SAFE: a randomized, double-blind safety trial of high dose nicotinamide riboside in Parkinson's disease. *Nat. Commun.* **14**, 7793 (2023). [10.1038/s41467-023-43514-6](https://doi.org/10.1038/s41467-023-43514-6), PMID: [38016950](http://www.ncbi.nlm.nih.gov/pubmed/38016950), PMCID: [PMC10684646](https://www.ncbi.nlm.nih.gov/pmc/articles/PMC10684646).

16. Presterud, R. et al. Long-term nicotinamide riboside use improves coordination and eye movements in ataxia telangiectasia. *Mov. Disord.* **39**, 360−369 (2024). [10.1002/mds.29645](https://doi.org/10.1002/mds.29645), PMID: [37899683](http://www.ncbi.nlm.nih.gov/pubmed/37899683).

17. Orr, M. E. et al. A randomized placebo-controlled trial of nicotinamide riboside in older adults with mild cognitive impairment. *GeroScience* **46**, 665−682 (2024). [10.1007/s11357-023-00999-9](https://doi.org/10.1007/s11357-023-00999-9), PMID: [37994989](http://www.ncbi.nlm.nih.gov/pubmed/37994989), PMCID: [PMC10828186](https://www.ncbi.nlm.nih.gov/pmc/articles/PMC10828186).

18. Pirinen, E. et al. Niacin cures systemic NAD+ deficiency and improves muscle performance in adult-onset mitochondrial myopathy. *Cell Metab.* 31, 1078–1090.e5 (2020). [10.1016/j.cmet.2020.04.008](https://doi.org/10.1016/j.cmet.2020.04.008), Pubmed: 32386566.

19. Ito, T. K. et al. A nonrandomized study of single oral supplementation within the daily tolerable upper level of nicotinamide affects blood nicotinamide and NAD^+^ levels in healthy subjects. *Translational Medicine of Aging* 4, 45–54 (2020). [10.1016/j.tma.2020.04.002](https://doi.org/10.1016/j.tma.2020.04.002).

20. Poyan Mehr, A. et al. De novo NAD+ biosynthetic impairment in acute kidney injury in humans. *Nat. Med.* **24**, 1351−1359 (2018). [10.1038/s41591-018-0138-z](https://doi.org/10.1038/s41591-018-0138-z), Pubmed:[30127395](https://www.ncbi.nlm.nih.gov/pubmed/30127395).

21. Takahashi, Y. et al. Nicotinamide suppresses hyperphosphatemia in hemodialysis patients. *Kidney Int.* **65**, 1099−1104 (2004). [10.1111/j.1523-1755.2004.00482.x](https://doi.org/10.1111/j.1523-1755.2004.00482.x), Pubmed:[14871431](https://www.ncbi.nlm.nih.gov/pubmed/14871431).

22. Irie, J. et al. Effect of oral administration of nicotinamide mononucleotide on clinical parameters and nicotinamide metabolite levels in healthy Japanese men. *Endocr. J.* **67**, 153−160 (2020). [10.1507/endocrj.EJ19-0313](https://doi.org/10.1507/endocrj.EJ19-0313), Pubmed:[31685720](https://www.ncbi.nlm.nih.gov/pubmed/31685720).

23. Pencina, K. M. et al. MIB-626, an oral formulation of a microcrystalline unique polymorph of β-nicotinamide mononucleotide, increases circulating nicotinamide adenine dinucleotide and its metabolome in middle-aged and older adults. *J. Gerontol. A Biol. Sci. Med. Sci.* **78**, 90−96 (2023). [10.1093/gerona/glac049](https://doi.org/10.1093/gerona/glac049), PMID: [35182418](http://www.ncbi.nlm.nih.gov/pubmed/35182418).

24. Yoshino, M. et al. Nicotinamide mononucleotide increases muscle insulin sensitivity in prediabetic women. *Science* **372**, 1224−1229 (2021). [10.1126/science.abe9985](https://doi.org/10.1126/science.abe9985), PMID: [33888596](http://www.ncbi.nlm.nih.gov/pubmed/33888596), PMCID: [PMC8550608](https://www.ncbi.nlm.nih.gov/pmc/articles/PMC8550608).

25. Katayoshi, T. et al. Nicotinamide adenine dinucleotide metabolism and arterial stiffness after long-term nicotinamide mononucleotide supplementation: a randomized, double-blind, placebo-controlled trial. *Sci. Rep.* **13**, 2786 (2023). [10.1038/s41598-023-29787-3](https://doi.org/10.1038/s41598-023-29787-3), PMID: [36797393](http://www.ncbi.nlm.nih.gov/pubmed/36797393), PMCID: [PMC9935856](https://www.ncbi.nlm.nih.gov/pmc/articles/PMC9935856).

26. Pencina, K. M. et al. Nicotinamide adenine dinucleotide augmentation in overweight or obese middle-aged and older adults: A physiologic study. *J. Clin. Endocrinol. Metab.* **108**, 1968−1980 (2023). [10.1210/clinem/dgad027](https://doi.org/10.1210/clinem/dgad027), PMID: [36740954](http://www.ncbi.nlm.nih.gov/pubmed/36740954).
